# Cytolethal distending toxin induces the formation of transient messenger-rich ribonucleoprotein nuclear invaginations in surviving cells

**DOI:** 10.1371/journal.ppat.1007921

**Published:** 2019-09-30

**Authors:** Lamia Azzi-Martin, Wencan He, Christelle Péré-Védrenne, Victoria Korolik, Chloé Alix, Martina Prochazkova-Carlotti, Jean-Luc Morel, Emilie Le Roux-Goglin, Philippe Lehours, Mojgan Djavaheri-Mergny, Christophe F. Grosset, Christine Varon, Pierre Dubus, Armelle Ménard

**Affiliations:** 1 Univ. Bordeaux, INSERM, Bordeaux Research in Translational Oncology, BaRITOn, U1053, Bordeaux, France; 2 Institute for Glycomics, Griffith University, Gold Coast Campus, Gold Coast, Australia; 3 Univ. Bordeaux, CNRS; UMR5293, Institut des maladies neurodégénératives, Bordeaux, France; 4 CHU de Bordeaux, Laboratoire de Bactériologie, Centre National de Référence des Campylobacters et des Hélicobacters, Bordeaux, France; 5 Univ. Bordeaux, INSERM U1218 ACTION, Institut Bergonié, Bordeaux France, INSERM UMRS1138, Centre de Recherche des Cordeliers, Paris, France. Metabolomics and Cell Biology Platforms, Institut Gustave Roussy, Villejuif, France; 6 Univ. Bordeaux, INSERM, Biothérapies des Maladies Génétiques, Inflammatoires et Cancer, BMGIC, U1035, Bordeaux, France; 7 CHU de Bordeaux, Pôle biologie et pathologie, Service de biologie des tumeurs, Bordeaux, France; University of Illinois, UNITED STATES

## Abstract

Humans are frequently exposed to bacterial genotoxins involved in digestive cancers, colibactin and Cytolethal Distending Toxin (CDT), the latter being secreted by many pathogenic bacteria. Our aim was to evaluate the effects induced by these genotoxins on nuclear remodeling in the context of cell survival. *Helicobacter* infected mice, coculture experiments with CDT- and colibactin-secreting bacteria and hepatic, intestinal and gastric cells, and xenograft mouse-derived models were used to assess the nuclear remodeling *in vitro* and *in vivo*. Our results showed that CDT and colibactin induced-nuclear remodeling can be associated with the formation of deep cytoplasmic invaginations in the nucleus of giant cells. These structures, observed both *in vivo* and *in vitro*, correspond to nucleoplasmic reticulum (NR). The core of the NR was found to concentrate ribosomes, proteins involved in mRNA translation, polyadenylated RNA and the main components of the complex mCRD involved in mRNA turnover. These structures are active sites of mRNA translation, correlated with a high degree of ploidy, and involve MAPK and calcium signaling. Additional data showed that insulation and concentration of these adaptive ribonucleoprotein particles within the nucleus are dynamic, transient and protect the cell until the genotoxic stress is relieved. Bacterial genotoxins-induced NR would be a privileged gateway for selected mRNA to be preferably transported therein for local translation. These findings offer new insights into the context of NR formation, a common feature of many cancers, which not only appears in response to therapies-induced DNA damage but also earlier in response to genotoxic bacteria.

## Author summary

Humans are frequently exposed to bacterial genotoxins linked to cancers, colibactin and Cytolethal Distending Toxin (CDT). These genotoxins induce DNA damage and can promote formation of nucleoplasmic reticulum (NR), deeply invaginated in the nucleoplasm of giant nuclei both *in vivo* and *in vitro*. Our cellular models showed that these structures can be observed together with profound nuclear reorganization corresponding to remodeling of nuclear material. The core of the genotoxin-induced NRs concentrates protein production machinery of the cell as well as controlling elements of protein turnover. These genotoxin-induced dynamic structures may also be signaling hubs controlling mRNA turnover and translation of selected mRNAs and thus correspond to a privileged gateway for the synthesis of selected mRNA which are preferentially transported from the nucleus through pores and translated therein. These transient and reversible hubs allow the cell to pause and repair the DNA damage caused by bacterial genotoxins in order to maintain cell survival. As NR formation is a common feature of many cancers, similar mechanism could occur and contribute to the resistance of cancer cells to radiotherapies and some chemotherapies aimed at inducing DNA damage.

## Introduction

Humans are frequently exposed to bacterial genotoxins associated with digestive diseases such as the cytolethal distending toxin (CDT) and colibactin, a polyketide-non-ribosomal peptide genotoxin. CDT had been identified in many pathogenic mucosal bacteria of the microbiota, *i*.*e*. *Campylobacter* species, *Helicobacter* species, *Escherichia* species, *Shigella* species, *Salmonella* species, *Haemophilus* species, *Aggregatibacter actinomycetemcomitans* and *Providencia alcalifaciens*. CDT is composed of 3 subunits, i.e. CdtA, CdtB and CdtC, of which CdtB subunit is the most conserved of the subunits among CDT-secreting bacteria (reviewed in [1] and [2]). The study of toxins produced by these bacteria revealed a conserved mechanism of action related to the active CDT subunit CdtB, a dual-function enzyme that can act as a phosphatidylinositol-3,4,5-trisphosphate phosphatase and a DNase. CdtB targets the nucleus where it induces single and double-strand DNA breaks, which is associated with a cell cycle arrest and a cellular and nuclear distention (reviewed in [3]). Similar effects are caused upon colibactin intoxication [4].

A direct correlation between the induction of DNA damage and invagination of the nuclear envelope was demonstrated using etoposide, a topoisomerase II inhibitor [5]. These structures, also called reticular membrane network, (intra)nucleoplasmic reticulum (NR) or nuclear tubules/invaginations, involve the relocation of the RNA binding protein, UNR/CSDE1 (Upstream of N-Ras/Cold shock domain-containing protein E1), that appear to be concentrated in cytoplasmic cores deeply invaginated into the distended nuclei, forming a novel type of NR, called “UNR-NR”, involved in mRNA translation [6]. Indeed, various NR are known to be induced in different physiological or pathological states [7]. Their appearance increases in tumor cells. Microorganisms also trigger the formation of NR and even hijack them to sustain efficient colonization, multiplication and survival [7]. However, NR formation had not been reported during bacterial infection. Only intranuclear pseudoinclusions were shown in mouse liver cells treated with *Helicobacter pullorum* sonicates [8]. Together, these observations suggested a possible link between CDT and NR formation that requires elucidation.

In this study, we used *in vivo* and *in vitro* models to investigate the role of bacterial genotoxins in nuclear remodeling. Liver tissues of mice infected with *Helicobacter hepaticus* were used first. Then, *in vitro* cocultures of human cell lines were performed using wild type strains of *H*. *hepaticus* and *H*. *pullorum*, their corresponding CDT-knockout mutant strains [9], as well as other toxin-secreting bacteria. To attribute the effects observed specifically to CDT, we used transgenic cell lines allowing the tetracycline-inducible expression of the active subunit CdtB of *H*. *hepaticus* and its corresponding H265L mutated CdtB (CdtB-H265L), lacking catalytic activity [10]. These transgenic cell lines were engrafted into immunodeficient mice and the xenograft-derived tumors were used to characterize the nuclear remodeling *in vivo*. Time course experiments of NR formation, DNA and RNA FISH, transmission electron microscopy, ribopuromycilation assay, UNR silencing and pharmacological inhibitors assays were conducted to characterize the genotoxin-induced UNR-NR. As UNR is a RNA-binding protein involved in cytoplasmic mRNA metabolism that was found in different RNA-associated complexes, its known partners have been evaluated using immunostaining/imaging.

### Ethics statement

Animal material provided from previous studies approved by the Ethics Committee for Animal Care and Experimentation CEEA 50 in Bordeaux (Comité d'Ethique en matière d'Expérimentation Animale agréé par le ministre chargé de la Recherche, “dossier no. Dir 13126B- V2”, “saisines” no. 4808-CA-I [11] and 13126B [10], Bordeaux, France), according to the treaty no.123 of the European Convention for the Protection of Vertebrate Animals. Animal experiments were performed in A2 animal facility (security level 2) by trained authorized personnel only.

## Materials and methods

Cell lines, bacterial strains and culture conditions are shown in Table 1. The content of NR is morphologically defined using immunostaining and electronic microscopy. UNR-NR was monitored using UNR protein as a marker [6]. Antibodies and the working dilutions used for immunohistochemistry and immunocytochemistry are detailed in S1 Table. Reagents and secondary antibodies, infection of mice with *H*. *hepaticus*, ribopuromycilation assay, RT-qPCR, and statistical analyses are detailed in Supplementary Materials and Methods.

**Table 1:** Bacterial strains and cell lines.

**Bacterial strain**	**Source**	**Virulence factors/phenotype (genotype)**	**Reference**	**Culture**
*H*. *pullorum* strain H495	CCUG 33840, 459–94, Burnens 459–94, H59-94	CDT^+^, T6SS^+^	[43]	[12]
*H*. *pullorum* strain H495 ΔCDT	INSERM U1053, Bordeaux, France	CDT^-^, T6SS^+^	[44]	[12]
*H*. *hepaticus* strain 3B1/Hh-1	Massachusetts Institute of Technology, Cambridge, US (James G Fox)	CDT^+^, T6SS^+^	[45]	[11]
*H*. *hepaticus* strain 3B1/Hh-1 ΔCDT		CDT^-^, T6SS^+^	[45]	[11]
*H*. *felis* strain CS1	ATCC 49179, Pasteur Institute (Agnès Labigne), Paris, France	T3SS^+^, CagA^-^, VacA^-^	[46]	[47]
*H*. *pylori* strain 7.13*	Vanderbilt University, Nashville, TN, US (Richard Peek)	T4SS^+^, CagA^+^, VacA^-^(*vacA s1a/m2*)*	[48]	[49]
*H*. *pylori* strain HPAG1	Karolinska Institute (Lars G. Engstrand), Stockholm, Sweden	T4SS^+^, CagA^+^, VacA^+^ (*vacA s1b/m1*)	[50]	[49]
*H*. *pylori* strain SS1**	Hyogo College of Medicine (Yoshihiro Fukuda), Hyogo, Japan	T4SS^-^, CagA^+^, VacA^-^ (*vacA s2/i2/m2*)	[51]	[49]
*H*. *pylori* strain TN2GF4	Hyogo College of Medicine (Yoshihiro Fukuda), Hyogo, Japan	T4SS^+^, CagA^+^, VacA^+^ (*vacA s1b/m1*)	[52]	[49]
*E*. *coli* strain DH10B BAC vector	IRSD—INRA Toulouse, France (Eric Oswald)	CDT^-^, colibactin^*-*^ (*pKS*^*-*^)	[4]	[4]
*E*. *coli* strain DH10B BAC pks		CDT^-^, colibactin^*+*^ (*pKS*^*+*^)	[4]	[4]
*E*. *coli* Shiga toxin-2 strain	Clinical strain from Bordeaux Hospital, France	CDT^-^, *stx1*^*-*^, *stx2*^*+*^, *eae*^*+*^	/	[53]
**Cell line**^**§**^**, type**	**Collection**	**Type**	**Origin, reference**	**Culture medium**^**§**^
AGS	ATCC CRL-1739	Epithelial gastric	human gastric adenocarcinoma	DMEM-F12
SW480 [SW-480]	ATCC CCL-228	Epithelial intestinal	human colon adenocarcinoma	DMEM
Hep3B (Hep 3B, Hep-3B), epithelial hepatic	ATCC HB-8064	Epithelial hepatic	human hepatocellular carcinoma	DMEM
Hep3B-RFP	INSERM U1053, Bordeaux, France	Epithelial hepatic	[10]	DMEM ± doxycycline
Hep3B-*Hh*-CdtB				
Hep3B-*Hh*-CdtB-H265L				

CagA^+^, protein encoded by the cytotoxin-associated gene A (*cagA*);

CDT^+^, expression of the cytolethal distending toxin (*cdtABC*) operon;

ΔCDT, CDT knockout strain;

Eae^+^, expression of the intimin;

Stx1^+^, expression of the Shiga toxin-1;

Stx2^+^, expression of the Shiga toxin-2;

T4SS^+^, type IV secretion system;

T6SS^+^, type VI secretion system;

VacA, Vacuolating cytotoxin A.

*Helicobacter pylori* and *H*. *felis* are non-CDT secreting *Helicobacter* species.

*Helicobacter pylori 7.13 strain is a single colony output derivative of *H*. *pylori* strain B128 recovered 3 weeks post-challenge of infected gerbils. This strain does not produce a detectable VacA protein due to the presence of a naturally occurring mutation in *vacA* leading to a truncated protein. This strain causes apoptosis and DNA damage in mouse, gerbil, and human gastric epithelial cells [48].

**Helicobacter pylori strain SS1 lacks a functional T4SS and contains a non-toxigenic vacA allele (s2/i2/m2).

Kanamycin (Sigma Aldrich France) (20 μg/ml) was added for the culture of the CDT knock-out strains.

*Escherichia coli* strains were routinely grown in Luria-Bertani medium at 37°C except for strains DH10B harboring the BAC vectors that were grown in Luria Bertani medium with chloramphenicol (25 μg/ml).

^$^Cell lines were verified by genotyping. They were maintained in culture medium supplemented with 10% heat-inactivated fetal calf serum (Invitrogen), 50 μg/ml of vancomycin (Sigma Aldrich France) and penicillin/streptomycin (100 u/mL each) at 37°C in a 5% CO_2_ humidified atmosphere. Antibiotics were removed 24 h prior bacterial infection and during coculture experiments.

Hep3B-derived transgenic cell lines were established by lentiviral transduction, as previously reported [10]. Cells having the integrated transgene sequence in a transcriptionally silent form were selected in the presence of puromycin (2 μg/ml). When required, the transgene expression was induced in the cells from the tetracycline-inducible promoter by addition of doxycycline (200 ng/ml) to the culture medium and incubation for 72 h.

BAC pks, functional pks island encoding colibactin

BAC, bacterial artificial chromosome

CDT, cytolethal distending toxin.

### Coculture experiments

Non-transgenic cells were seeded on culture plates or glass coverslips 24 h before addition of bacteria to the culture media at a density appropriate for each experiment (20,000 to 40,000 cells per well for glass coverslips). *H*. *hepaticus* and *H*. *pullorum* suspensions were prepared in Brucella broth to optical density of 0.6 (600 nm) corresponding to a concentration of 2.8 x10^8^ colony forming units/ml; *H*. *pylori* suspensions were prepared in Brucella broth to optical density of 1.0 (600 nm) corresponding to a concentration of 2.1x10^8^ colony forming units/ml; *E*. *coli* suspensions were prepared in Luria Bertani medium to optical density of 1.0 (600 nm) corresponding to a concentration of 5x10^8^ colony forming units/ml. For coculture experiments, the culture medium was removed and a volume corresponding to a multiplicity of infection (MOI) of 100 bacteria/cell in renewed medium supplemented with fetal calf serum was added and incubation continued for 72 h. For some coculture experiments, bacteria were seeded on semipermeable tissue culture inserts (0.2 μm pore size, Anopore, Nunc, Naperville, IL, USA) fitted into wells containing cultured epithelial cells. Cells (Table 1) were either not infected to serve as controls or infected for 72 h with *H*. *hepaticus*, *H*. *pullorum* and *E*. *coli* at a multiplicity of infection (MOI) of 100 bacteria/cell as previously reported [4]. For *H*. *pylori*, infections were carried out for 24, 48 and 72 h at a MOI of 25 bacteria/cell [12].

### UNR immunodetection on tissue sections combined with interphase fluorescence *in situ* hybridization (FISH)

To correlate UNR association with the chromosomal content (DNA FISH) or with the poly(A) tail of mRNA (RNA FISH), sequential fluorescent labeling was performed using 6-μm tissue sections, prepared from formalin-fixed paraffin-embedded human Hep-3B xenografts [10]. Firstly, UNR immunodetection was performed, images were captured and coordinates of each image were recorded to ensure repositioning of the slide to the same area after FISH assay. Secondly, interphase DNA or RNA FISH detection was performed using the same slide; the images were captured using the repositioning function of the microscope (Zeiss Axioplan 2 fluorescence microscope, Zeiss, Jena, Germany).

DNA FISH targeted the short arm of chromosome 6 (chromosome region 6p25) (SpectrumRed probe, Abbott France, Rungis, France) and the long arm of chromosome 11 (chromosome region 11q13.3) (SpectrumGreen probe, Abbott France) and was performed as previously described [13]. RNA FISH targeting polyadenylated mRNA was also performed as described [6]. At each step, slides were mounted with Vectashield antifade medium containing 4’,6-diamidino 2-phenylindole (DAPI) (Vector Laboratories, Laboratoires Eurobio/Abcys, Les Ulis, France).

### Small interference RNA silencing

siRNA silencing was performed using a pool of 3 target-specific 19–25 nucleotide siRNAs designed to knock down UNR gene expression (sc-76809, Santa Cruz Biotechnology, Heidelberg, Germany). Transgenic Hep3B cells (Table 1) were grown in 12-wells cell culture plates to 70% confluency and the transgene expression was induced with doxycycline (200 ng/ml) 18 hours prior to siRNA transfection according to optimized conditions. As a result, 20 pMoles siRNAs anti-UNR or a scrambled control sequence siRNA (5’-GGGCAAGACGAGCGGGAAG-3’) was added and mixed with lipofectamine (Lipofectamine RNAiMAX Transfection Reagent, Invitrogen, Carlsbad, CA, US) in Opti-MEM medium according to manufacturer’s instructions.

### Immunofluorescence, image analysis and protein quantification

Cell cultures grown on glass coverslips and fluorescently labeled were mounted on microscope slides with Fluoromount-G (Clinisciences SA, Montrouge, France) and treated as previously reported [12] with minor modifications. Tissue sections prepared from formalin-fixed paraffin-embedded tissues were also submitted to immunofluorescence protocol. Proteins were analyzed by immunostaining with primary antibodies (reference and dilutions are shown in S1 Table). Dual-, triple- and quadruple-color imaging with Alexa Fluor 647-labeled (far-red), Alexa Fluor594-labeled or Fluor 594-labeled phalloidin (red), Alexa Fluor 488-labeled (green) secondary antibodies, and 4',6'-diamidino-2-phenylindole (DAPI, blue) was obtained using selective laser excitation at 647, 594, 488 and 358 nm, respectively. Traditional widefield fluorescence imaging was performed using Eclipse 50i epi-fluorescence microscope (Nikon, Champigny sur Marne, France) equipped with the Nis Element acquisition software and a 640 (numerical aperture, 1.3) oil immersion objective (40X). Confocal microscopy was performed as previously reported [9] using a SP5 confocal microscope (Leica, Leica microsystems GmbH, Wetzlar, German) with a ×63/numerical aperture 1.4 Plan Neofluor objective lens. To prevent cross-contamination between fluorochromes, each channel was imaged sequentially by using the multitrack recording module before merging. z-stack pictures were obtained using LAS AF, Leica software. Subsequent quantification of proteins in nucleoplasm, cytoplasm and NR were performed with ImageJ (v. 1.52n) [14].

Protein quantification in nucleoplasm, cytoplasm and foci was performed by measuring the pixel intensity with the “Plot Profile” function of ImageJ (v. 1.52n) [14] using capture of fluorescent staining (confocal imaging). For cytoplasmic protein, the quantification data from the nucleoplasm corresponding to background noise have been substracted from those of the cytoplasm and foci.

The number of cells with UNR-positive nuclei *in vivo* (formalin-fixed paraffin-embedded tissue) might be underestimated compared to that of cells with a smaller nucleus, as 3 μm- and 6 μm-tissue sections were cut and the size of the nuclei is significantly increased in response to the CDT.

## Results

### *Helicobacter hepaticus* infection triggers nuclear remodeling of hepatocytes in mice in association with the formation of UNR nucleoplasmic reticulum *in vivo*

**Fig 1:**
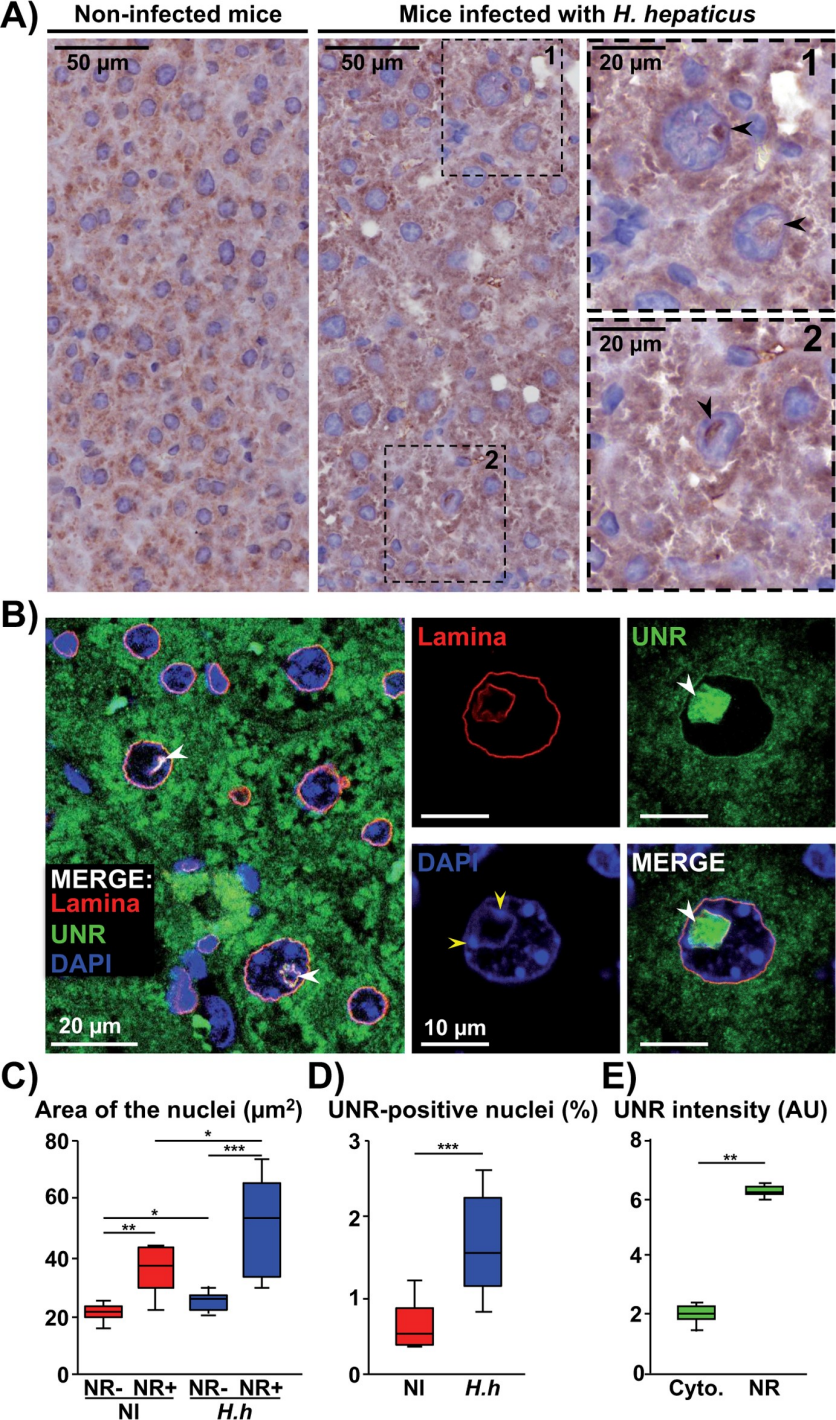
*In vivo* detection of UNR protein in liver of mice infected with *Helicobacter hepaticus*. Non-transgenic mice were infected with *H*. *hepaticus* wild type strain 3B1for 14 months [11]. Images of mouse livers following a 14 months infection with *H*. *hepaticus*. **(A)** Three μm-tissue sections of liver specimens immunostained for UNR and counterstained with standard hematoxylin staining. Magnifications of selected areas are shown in boxes. Black arrowheads indicate UNR-NR. **(B)** Confocal image of tissue sections of infected liver stained with fluorescent primary and secondary antibodies: the nuclear lamina (red), UNR (green) and DAPI to counterstain the nucleus (blue). White and yellow arrowheads indicate UNR-NR and areas where lamina and chromatin (DAPI) are connected, respectively. **(C)** Nuclear area and **(D)** UNR-positive nuclei, representative of 3,000 cells per mice. (**E)** UNR protein quantification in cytoplasm *vs* NR. For panels (C) to (E), nuclear surface was quantified by isolating the DAPI fluorescence for each nucleus by using the ‘Threshold’ function of ImageJ (v. 1.52n). The acquired images were calibrated according to the microscope software manufacturer. A minimum of 1,000 nuclei were measured. The number of nuclei gave the number of cells for each image. The percentage of cells presenting UNR-NR was determined by manually counting the number of nuclei displaying UNR spots in the nucleoplasm. UNR intensity was measured by using the ‘Plot Profile’ function of ImageJ [54], which consists in drawing a line that crosses the cytoplasm and a UNR-NR and measuring the pixel intensity along the drawn line. * p = 0.0288, ** p = 0.002 and *** p<0.0001 AU, arbitrary units; Cyto., cytoplasm; DAPI, 4′, 6′-diamidino-2-phenylindol; *H*.*h*, *Helicobacter hepaticus;* NI, non-infected; NR, nucleoplasmic reticulum.

Immunohistochemical analyses of the liver of mice infected with *H*. *hepaticus* for 14 months [11] revealed the localization of UNR in nuclear foci of some giant hepatocytes (Fig 1A). Immunofluorescent staining confirmed the remodeling of hepatocytes following *H*. *hepaticus* infection with enlarged nuclei (Fig 1B and 1C). Quantification analysis revealed that nuclear foci formation was a rare event in non-infected mice while a 3-fold increase of NR+ cells was detected in livers of infected mice (Fig 1D), with an average surface area of 50.6 μm^2^ for these NR+ nuclei *vs* 25.5 μm^2^ for NR- nuclei in infected mice (Fig 1C, blue boxes), corresponding to 270 μm^3^ *vs* 96.9 μm^3^. The nuclear foci were found to lack DAPI staining, concentrated UNR protein (3.06 ± 0.47-fold increase in nuclear foci *vs* the cytoplasm, p = 0.002, Fig 1E) and were surrounded by the nuclear lamina and DAPI foci (yellow arrowheads, Fig 1B).

NR were not detected in tumoral tissue of the liver in *H*. *hepaticus*-infected mice presenting hepatocarcinoma, but rather in the neighboring non-tumoral areas or in mice without adenocarcinoma (S1 Fig). This suggested that *H*. *hepaticus* is directly responsible for NR formation in mice. Moreover, obvious formation of UNR-NR in the giant cells indicates that CDT of *H*. *hepaticus* may play a role in the formation of these structures.

Stomachs of mice infected with the non-CDT secreting *H*. *felis* strain CS1 for 55 weeks [15] were also analyzed. Immunohistochemical analyses did not reveal any UNR-NR formation in non-infected nor infected mice (S2 Fig). Similar results were obtained when using stomachs of mice infected with *H*. *pylori* strains SS1, HPAG1 and TN2GF4 for 55 weeks [15], regardless of the virulence factors secreted by these *H*. *pylori*. These results further correlate with the absence of CDT secretion by these gastric Helicobacters.

Together these data suggest that the invaginated structures observed in the hepatocytes of mice infected with *H*. *hepaticus* exhibit characteristics of the previously reported “UNR-NR” [6].

### Infection with genotoxin-secreting bacteria promotes formation of nucleoplasmic reticulum *in vitro*

**Fig 2:**
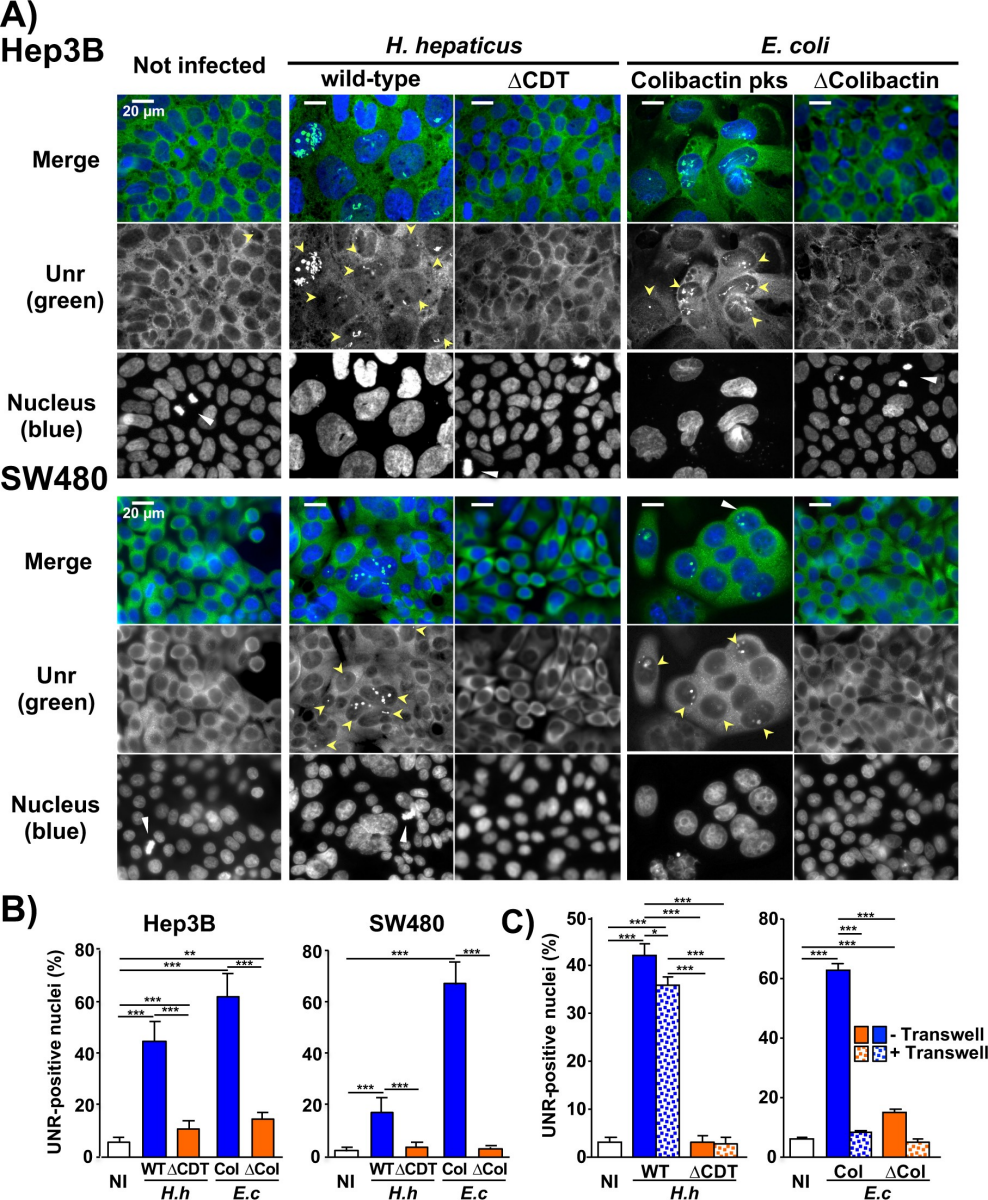
*In vitro* detection of UNR protein during bacterial infection. **(A**) Images of liver Hep3B and colon SW480 cells following a 72h coculture. Cells were stained with fluorescent primary and secondary antibodies targeting UNR (green) and DAPI to counterstain the nucleus (blue). Fluorescent staining was observed using wide field fluorescence imaging [44]. Yellow and white arrowheads indicate UNR-NR and cells undergoing mitosis, respectively. **(B)** Quantification of UNR-NR positive nuclei (%). At least 200 cells were counted for each experiment. Data represent the mean of triplicates in 1 representative experiment out of 3. ** p = 0.0003 and *** p<0.0001. **(C)** Coculture experiments with liver Hep3B were also performed using a 0.4-μm Transwell system. * p<0.0154 and *** p<0.0001 ΔCDT, CDT-knockout mutant strain; ΔCol., bacterial artificial chromosome; Col., pks genomic island encoding colibactin; DAPI, 4′, 6′-diamidino-2-phenylindol; *E*.*c*., *Escherichia coli; H*.*h*., *Helicobacter hepaticus;* NI, non-infected; WT, wild-type *H*. *hepaticus* strain 3B1.

Coculture experiments with *H*. *hepaticus* and *H*. *pullorum* were performed with Hep3B hepatic cells and SW480 intestinal cells (Table 1), as these bacilli colonize the intestine and the liver. Both Helicobacters induced a significant increase of UNR-NR within the larger nuclei of both cell types (Figs 2 and S3A and S3B). The formation of UNR-NR was almost absent in non-infected cells and in cells infected with the CDT-knockout mutant strains, suggesting that these two CDT-secreting Helicobacters are associated with the formation of nuclear UNR-NR and that the CDT is likely to be the main virulence factor associated with UNR-NR formation. NR formation was also observed in Huh7 hepatic and HT29 intestinal cells where tiny UNR-rich foci were more rarely observed.

Non-CDT secreting bacterial pathogens *H*. *pylori* and Shiga toxin-2 secreting *E*. *coli* did not induce major nuclear remodeling nor increased the number of UNR-NR in Hep3B, SW480 and in the *H*. *pylori*-susceptible AGS gastric cell line (Table 1 and S3A–S3D Fig and Supplementary Results). In contrast, those cell lines showed a significant nuclear remodeling associated with the increase in UNR-NR upon infection with CDT-secreting Helicobacters or colibactin-secreting extra-intestinal pathogenic *E*. *coli* (Figs 2 and S3A–S3C). Taken together, these results suggest that CDT and colibactin, two bacterial cyclomodulins and genotoxins, induced the formation of UNR-NR.

Coculture experiments were also performed with Hep3B cells using a Transwell system which prevents contact between bacteria and cultured cells, but allows the diffusion of soluble factors. NR formation was still observed after coculture with *H*. *hepaticus* in the Transwell system, but to a lesser extent, while colibactin-induced NR formation was not observed (Fig 2C). These results present additional support for attributing NR formation to these toxins. Indeed, CDT is internalized in the host cell by endocytosis independently of the contact with the host cell, while colibactin is directly injected into the host cell during *E*. *coli* infection, which requires direct contact with the host cell [4].

### The CdtB subunit promotes the nuclear remodeling and UNR nucleoplasmic reticulum formation in hepatocytes

**Fig 3:**
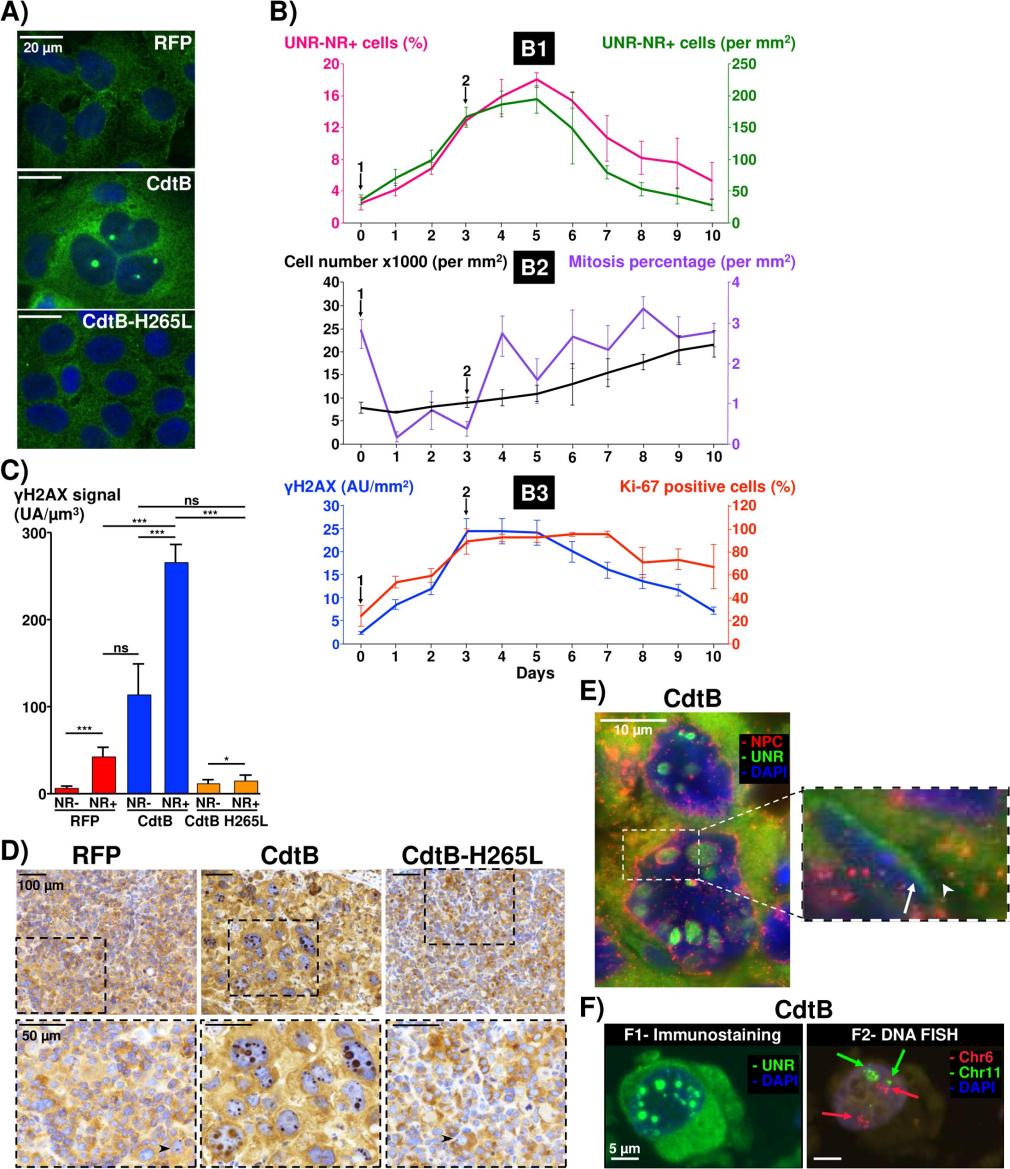
*In vitro* effects of the CdtB subunit on the localization of UNR protein in liver transgenic cell lines. **(A)** Images of hepatic Hep3B transgenic cells expressing the control Red Fluorescent Protein (RFP), the CdtB of *H*. *hepaticus* strain 3B1 (CdtB) or the CdtB of *H*. *hepaticus* strain 3B1 with the H265L mutation (CdtB-H265L). Cells were stained with fluorescent primary and secondary antibodies targeting UNR (green) and DAPI to counterstain the nucleus (blue). **(B)** Hep3B transgenic cells were cultivated with doxycycline for 72 h (arrow 1). Then doxycycline was removed and new medium was added (arrow 2). Quantification of UNR-NR-positive cells was performed daily until 10 days. **(B1)** The percentage of UNR-NR-positive cells was determined, as well as the number of UNR-NR-positive cells per mm^2^. **(B2)** The number of cells per mm^2^ and the percentage of mitotic cells was also determined. **(B3)** γH2AX foci intensity and the percentage of Ki-67-positive cells were quantified. Data represent the mean of triplicates in 1 representative experiment out of 3. **(C)** Hep3B transgenic cells were cultivated with doxycycline for 72 h. Cells were stained with fluorescent primary and secondary antibodies targeting γH2AX and UNR, and DAPI to counterstain the nucleus. Quantification of γH2AX foci intensity in UNR-NR-positive and -negative nuclei was performed. At least 200 cells were counted for each experiment. Data represent the mean of triplicates in 1 representative experiment out of 3. * p<0.05 and *** p<0.001. **(D)** Images of 3 μm-tissue sections of Hep3B-CdtB-derived mice engrafted tumors immunostained for UNR and counterstained with standard hematoxylin staining. Arrowhead indicates tiny NR. **(E)** Images of 3 μm-tissue sections of Hep3B-CdtB-derived mice engrafted tumors stained with fluorescent primary and secondary antibodies: Nuclear Pore Complex Proteins (NPC, red), UNR (green) and DAPI to counterstain the nucleus (blue). Arrowhead and long arrow indicate NPC and the absence of UNR immunostaining within the UNR-rich foci, respectively. **(F)** Images of 6 μm-tissue sections of Hep3B-CdtB-derived mice engrafted tumors processed for fluorescent staining with primary antibodies to target UNR (green, **F1**) followed by DNA FISH experiments **(F2)** with labeled probes hybridizing to the band on the short arm of human chromosome 6 (red) and the long arm of chromosome 11 (green). Images (UNR/DAPI) were captured using the microscope that records the coordinates of each image, which allows repositioning on the same area after DNA FISH assay. Each fluorescent spot corresponds to one copy of the chromosome region. Nuclei were counterstained with DAPI (blue). Red and green arrows in (F2) indicate chromosome 6 and 11 foci, respectively. Fluorescent staining were observed using traditional wide field in (A), (E) and (F). Of note, the visibility of UNR-NR structures in (D) and (E) depends on the cutting orientation of the nucleus section. Magnifications of selected areas are shown in boxes. AU, arbitrary units; CdtB-H265, *H*. *hepaticus* CdtB with H265L mutation; CdtB, CdtB of *H*. *hepaticus* strain 3B1; Chr, Chromosome; DAPI, 4’,6-diamidino 2-phenylindole; NPC, Nuclear Pore Complex Proteins; ns, non-significant; RFP, red fluorescent protein.

To elucidate if the observed effects could be specifically attributed to the CdtB subunit, stable transgenic cell lines conditionally expressing the active CdtB [10] were used (Table 1). CdtB of *H*. *hepaticus* was expressed *in situ* in the cells in a time-dependent manner, controlled by doxycycline induction. *In vitro* analysis (Fig 3A) demonstrated the formation of UNR-NR in distended CdtB-expressing Hep3B cells as compared to the Red Fluorescent Protein (RFP)-expressing control cells and cells expressing the *H*. *hepaticus* H265L CdtB mutant, suggesting that this catalytic residue is critical for the induction of UNR-NR. The apparent increase in UNR in the cytoplasm observed using wide field microscopy in CdtB expressing cells (Fig 3A) is likely to be the consequence of an increase in the size of the cell leading to this optical effect, since UNR expression was similar in the 3 cell populations (S3E Fig).

Time course experiment showed that UNR-NR formation in Hep3B cells began as early as 24 h after CdtB induction with a maximum effect observed in 3 to 5 days (Fig 3B1), despite termination of the induction of CdtB on day 3. After 5 days, the percentage of UNR-NR-positive cells started to gradually decrease (Fig 3B1, pink curve) and a concomitant resumption of cellular proliferation and mitosis was observed (Fig 3B2, black and purple curve, respectively). It is therefore likely that the cells, having efficiently repaired their genome after CdtB-mediated DNA damage, proliferated *de novo* and biased the real percentage of UNR-NR-positive cells present on the coverslip. To further strengthen these data, the number of UNR-NR-positive cells per mm^2^ was quantified (Fig 3B1, green curve): a decrease of UNR-NR-positive cells was observed over time (from day 5 to 10) without any cell death, suggesting that CdtB-induced NR is a transient and reversible process.

UNR-NR formation was also associated with an increase of Ki-67 nuclear antigen (Fig 3B3, red curve). As Ki-67 is expressed during all active phases of the cell cycle, G1, S, G2, and mitosis, this increase most likely reflects the expression of Ki-67 accumulated in the G2 phase following the CdtB-induced G2/M cell cycle arrest, as previously reported [10]. Non-cycling cells arrested in G2/M were also previously reported to be Ki-67-positive in other studies [16][17]. Thus, Ki-67 is unlikely to be a pertinent marker for evaluation of cell proliferation in response to CDT intoxication.

UNR-NR was associated with γH2AX foci formation (Fig 3B3, blue curve), a surrogate marker for double-stranded DNA breaks, this effect being very important in response to the CdtB (Fig 3C). In addition, the stronger γH2AX signal correlated with the bigger nuclei (S3F Fig), confirming that CdtB-induced DNA damage is associated with megalocytosis.

Hep3B transgenic cells were also engrafted into immunodeficient mice subsequently treated with doxycycline to induce the transgene expression [10]. Immunostaining analysis of engrafted cells from the sacrificed animals showed highly distended nuclei (reaching sometimes 50 μm of diameter) with large and multiple UNR-NR in response to the CdtB expression as compared to RFP- and CdtB-H265L-derived cells which presented very few and tiny UNR-NR (Fig 3D). The fluorescence imaging confirmed that CdtB induced UNR-NR foci, reaching up to 10 μm, contained UNR proteins and were surrounded by proteins of the nuclear pore complex (NPC, Fig 3E). A zone without UNR immunostaining was observable in the center of some UNR-NR suggesting the existence of unidentified elements in the invaginated structures (long arrow, Fig 3E). A global decrease in DAPI staining, likely to reflect chromatin decondensation, was also observed in UNR-NR-containing nuclei of CdtB-expressing engrafted cells (S3G Fig).

As CdtB induced-NR occurred in the largest nuclei (Figs 1C and 3D), a possible link between NR formation and polyploidy was evaluated by DNA FISH. Subsequently, NR foci were identified in nuclei with a high number of chromosome (>8 copies) in more than 70% of the distended Hep3B-intoxicated cells. It is noteworthy that among enlarged cells, cells with higher chromosome content corresponded to those with the largest UNR-NR (Fig 3F).

### Effects of UNR silencing on CdtB-induced nucleoplasmic reticulum formation in hepatic cells

**Fig 4:**
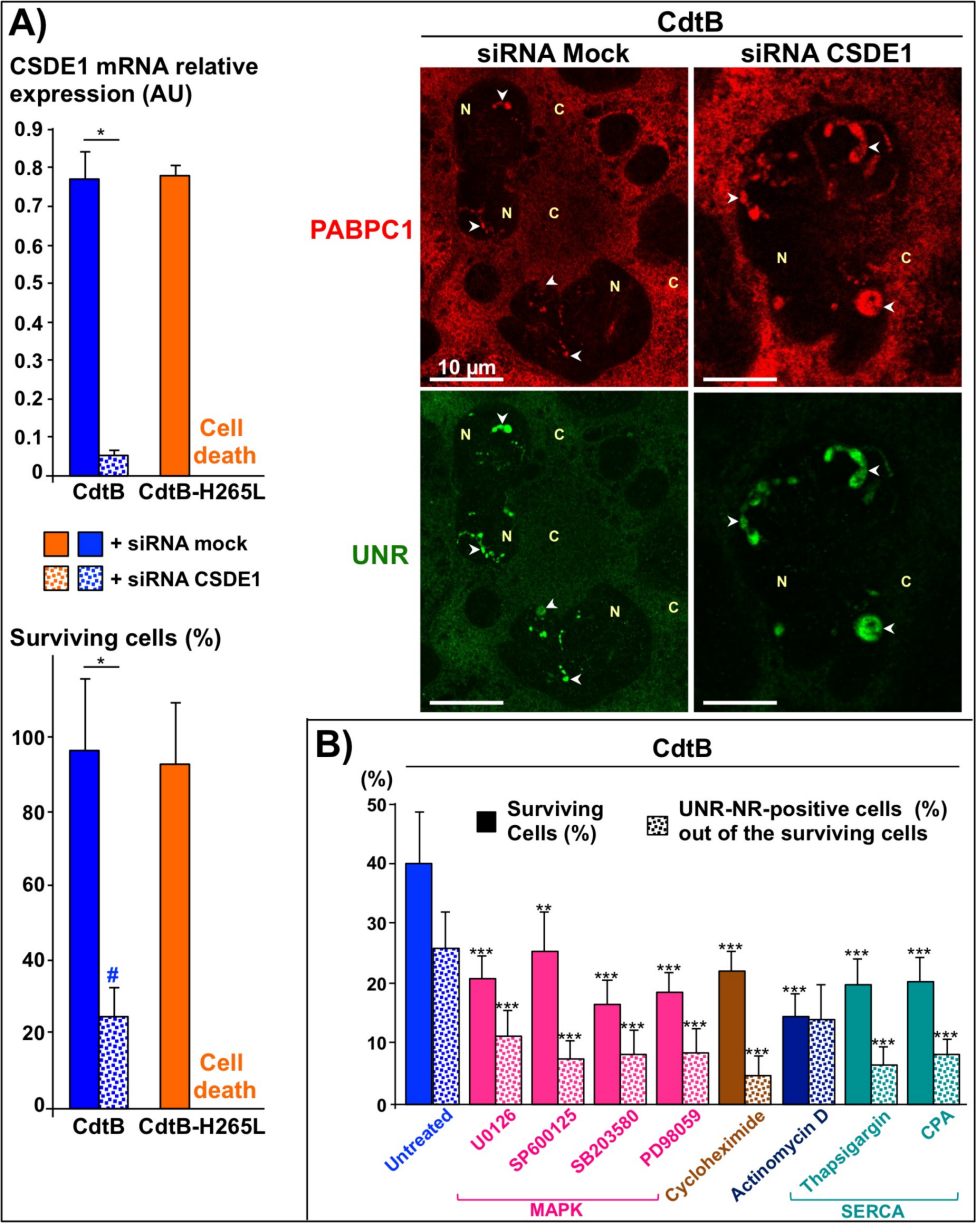
Effects of UNR silencing and pharmacological inhibitors on nucleoplasmic reticulum formation. **(A)** Hepatic Hep3B transgenic cells were transfected with siRNAs and concomitantly cultivated with doxycycline for 72 h to induce the expression of the control Red Fluorescent Protein (RFP), the CdtB of *H*. *hepaticus* strain 3B1 or the CdtB of *H*. *hepaticus* strain 3B1 with the H265L mutation (CdtB-H265L). Cells were stained with fluorescent primary and secondary antibodies targeting PABPC1 (red), UNR (green) and DAPI to counterstain the nucleus (blue). Fluorescent staining was observed using confocal fluorescence imaging [44]. Only CdtB surviving cells are shown. Arrowheads indicate UNR-NR. Expression of CSDE1 gene encoding the UNR protein was measured by real time quantitative RT-qPCR. Viable cells were counted by a direct plate count. Results are the means of three independent experiments, each performed in triplicate. # All of the UNR-depleted CdtB-surviving cells are UNR-NR+. * p<0.05. **(B)** Hepatic Hep3B transgenic cells were cultivated with doxycycline for 72 h. Inhibitors, *i*.*e*. U0126 (100 nM), SP600125 (100 nM), SB203580 (100 nM), PD98059 (100 nM), cycloheximide (1 μM), actinomycin D (0.7 μM), thapsigargin (100 nM) and cyclopiazonic acid (50 nM) were added to the medium either 24 h or 48 h after doxycycline induction, leading to similar results. Only the 24 hours-experiment is presented in this figure. Quantification of UNR-NR positive nuclei (%) was performed on a minimum of 500 cells. Data represent the mean of triplicates in 1 representative experiment out of 3. ** p<0.005 and *** p<0.0002. AU, arbitrary units; C, cytoplasm; CdtB-H265L *H*. *hepaticus* CdtB with H265L mutation; CdtB, CdtB of *H*. *hepaticus* strain 3B1; CPA, cyclopiazonic acid; MAPK, mitogen-activated protein kinase; N, nucleus; SERCA, sarco-endoplasmic reticulum Ca^2+^-ATPase.

siRNA-mediated silencing experiments were performed to examine the involvement of UNR in the formation of NR using Hep3B transgenic cell lines *in vitro*. Transfection with siRNAs (control and UNR/CSDE1 siRNA), carried out 24 hours after transgene induction, did not affect the cellular viability nor the formation of NR. Silencing UNR by adding the siRNA 12 h prior to transgene induction resulted in a massive cell death of RFP-, CdtB- and CdtB-H265L-expressing Hep3B cells, demonstrating that UNR is essential for cell survival. Transfection with siRNAs concomitantly with the transgene induction led to cell death of RFP- and CdtB-H265L-expressing cells, whereas CdtB-expressing giant cells displaying UNR-NR survived. RT-qPCR confirmed UNR/CSDE1 siRNA-mediated silencing (Fig 4A) in surviving cells, which displayed cytoplasmic UNR extinction, but still contained concentrated UNR in NR (Fig 4A). These data suggest that NRs enable the cells to resist interference-RNA mediated protein depletion, implicating NR formation in cell survival.

### Regulation of CdtB-induced nucleoplasmic reticulum formation

The effect of a range of pharmacological inhibitors on UNR-NR formation had been tested (Fig 4B). A reduction of UNR-NR formation was observed in presence of calcium channel blockers and mitogen-activated protein kinase (MAPK) inhibitors such as extracellular signal–regulated kinases (ERK1/2), c-Jun N-terminal kinase (JNK), and p38 MAPK. Furthermore, protein synthesis appears to play an important role in UNR-NR formation since cycloheximide reduced their formation. Actinomycin D, a transcription inhibitor, did not have a significant effect on NR formation.

### Characterization of CdtB-induced nucleoplasmic reticulum content in hepatic cells

**Fig 5:**
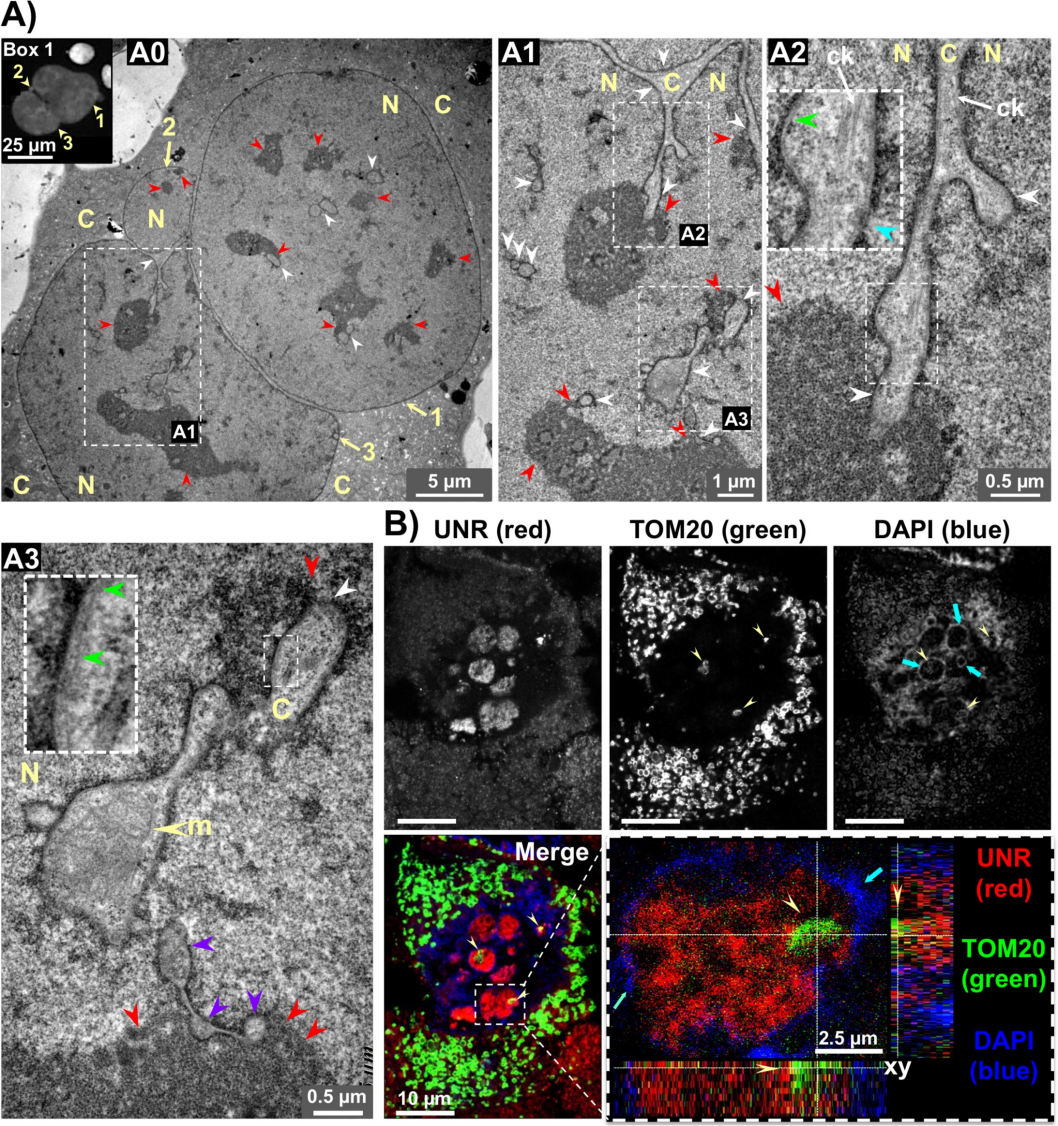
*In vitro* ultrastructure analysis of Hep3B-intoxicated cells. **(A)** Cross-sectional transmission electron micrograph of a Hep3B transgenic cell expressing the CdtB of *H*. *hepaticus*. A high-magnification transverse section from a cell presented a distended nucleus (diameter approx. 36 μm) having a trilobal appearance, as shown by the 3 yellow arrows, with a micronucleus like-structure (arrow no. 2) is shown in A0. Box 1 shows a DAPI staining of a Hep3B-intoxicated cell with a similar shape (wide field fluorescence imaging). White and red arrowheads indicate the cytoplasmic cores and nucleoli, respectively. Successive magnifications of **A0** are presented in **A1**, then in **A2** and **A3**. (A1) presents a long tubular channel formed by the invagination of the nuclear envelope that is continuous with the cytoplasm and extend into the nucleoplasm. In (A2) a nuclear invagination penetrating into a nucleolus is shown. The core contains cytoskeletal elements (white long arrow). The double-membraned wall is seen along the channels. A high-magnification of the inner and outer nuclear membranes with an intermembrane space (green arrowheads) is presented in boxes in (A2) and (A3). Blue arrowhead indicate nuclear pores in (A2). Some channels showing an association with nucleoli or terminating adjacent to nucleoli are also shown in (A2) and (A3). Some cytoplasmic cores are interconnected within the same nucleus (purple arrowheads in A3). Yellow arrowheads in A3 point to a mitochondrion inside a cytoplasmic core. **(B)** Confocal imaging of a 3 μm-tissue sections of Hep3B-CdtB-derived mice engrafted tumors stained with fluorescent primary and secondary antibodies: UNR (red), the mitochondrial import receptor subunit TOM20 (green) and DAPI to counterstain the nucleus (blue). Enlargement of the box represents xy slices of the middle of the z-stack and the projections of the orthogonal sections (dotted white lines) of the z-stack at the bottom and the right sides of each image. Yellow arrowheads point to the location of mitochondria in the corresponding core of UNR-NR surrounded by condensed DNA revealed by an intense DAPI labeling around the foci (blue arrows). White and red arrowheads indicate the cytoplasmic cores and nucleoli, respectively. C, cytoplasm; CdtB, CdtB of *H*. *hepaticus* strain 3B1; ck, cytoskeletal element; DAPI, 4′, 6′-diamidino-2-phenylindol; m, mitochondrion; N, nucleus.

**Fig 6:**
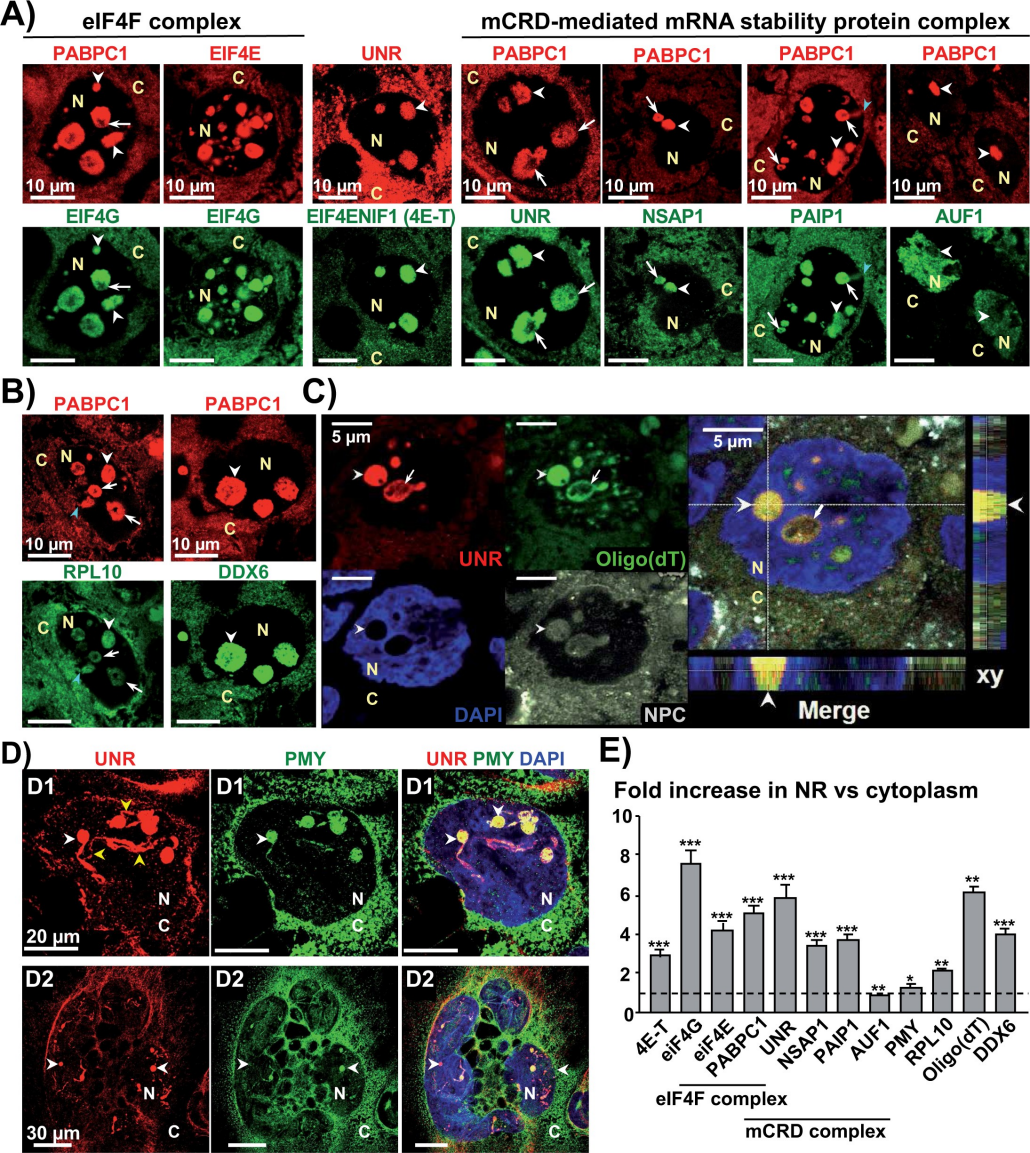
CdtB-induced Type-II nucleoplasmic reticulum concentrates mCRD-associated proteins, mRNA and are active site of translation. **(A)** Confocal images of 3 μm-tissue sections of Hep3B-CdtB-derived mice engrafted tumors immunostained for some eiF4F complex-associated proteins and the 5 subunits of the major coding region instability determinant (mCRD)-mediated mRNA stability complex. **(B)** Confocal images of 3 μm-tissue sections of Hep3B-CdtB-derived mice engrafted tumors stained with fluorescent primary and secondary antibodies: PABPC1 (red) and the ribosomal protein RPL10 (green) or DDX6 (green) and DAPI to counterstain the nucleus (blue). **(C)** Detection of messenger RNAs polyadenylation combined with tissue immunolabeling (6 μm-tissue section). Confocal image of 6 μm-tissue sections of Hep3B-CdtB-derived mice engrafted stained with fluorescent primary and secondary antibodies: UNR (red) and NPC (grey) and combined with FISH using an FITC-oligo (dT) probe (green) to detect poly(A) RNA and DAPI to counterstain the nucleus (blue). Enlargement of the box represents xy slices of the middle of the z-stack and the projections of the orthogonal sections (dotted white lines) of the z-stack at the bottom and the right sides of each image. **(D)** Confocal images of Hep3B transgenic cells expressing the CdtB of *H*. *hepaticus* analyzed using the ribopuromycilation assay to detect active translation. Cells were stained with fluorescent primary and secondary antibodies: UNR (red), puromycylated ribosome-bound nascent chains (translating ribosomes, PMY in green) and DAPI to counterstain the nucleus (blue). The picture presents a transverse section of a distended cell **(D1)** as well as a giant cell having a multilobal nucleus **(D2)**. **E** Protein quantification was performed on 100 NRs. The relative expression rate of protein in NR in response to the CdtB was reported as fold increase *vs* the expression in the cytosol. The discontinuous line shows the basal rate in cytoplasm. For some labeling, the simultaneous detection of different proteins couldn’t be performed due to similar host origin of the antibodies. PABPC1 was thus used instead of UNR to monitor the formation of nucleoplasmic reticulum, as they systematically colocalize. White arrowheads indicate the core of NR. Blue arrowheads in (A) and (B) indicate nuclear invagination connected to the cytoplasm. Yellow arrowheads in (D) indicate branched cores of NR. As in Fig 3D, NR showed uneven staining with stainless zones in the invaginated core (white arrows), suggesting the presence of mitochondria (as shown in Fig 5B). * p = 0.0230, ** p = 0.0012 and *** p<0.0001 C, cytoplasm; DAPI, 4′, 6′-diamidino-2-phenylindol; N, nucleus; NPC, Nuclear Pore Complex Proteins.

Classification of NR structures has been proposed according to the involvement of the outer nuclear membrane [7]. Most CdtB-induced invaginations corresponded to Type II double-membrane-walled invagination of the inner and outer nuclear membranes with an inter-membrane space (green arrowheads, Fig 5A2 and 5A3). This double-membraned NR was continuous with the cytoplasm (Fig 5A0), pierced by nuclear pores (blue arrowhead, Fig 5A2) and enclosed a cytoplasmic core that extended into the nucleoplasm (Fig 5A0). Some cores were interconnected within the same nucleus (Figs 5A3 and 6D1). Nuclear lamina and NPC were detected around the cytoplasmic core (Figs 1B and 3E and S4). Some invaginations terminated as blind-ending tubes within the nucleoplasm, the nucleoplasmic ends showing an association with nucleoli (Fig 5A, red arrowhead). NR contained ribosomes (Fig 6B), cytoskeletal elements (Fig 5A2, white long arrow) and mitochondria (Fig 5A3 and 5B). The zone without UNR staining in the core, observed in Figs 3E and 6A, 6B and 6C, probably corresponds to a mitochondrion. As previously observed [18], NR contain calnexin, a molecular chaperone of the endoplasmic reticulum (ER), and multi-pass ER membrane receptors for inositol 1,4,5-trisphosphate (S4 Fig). Other cytoplasmic proteins were also found in NR, most of these, however, were present at the same level as in the cytoplasm (S4 Fig). No association between NR formation and senescence could be found (S3H Fig). CdtB-induced NR appeared to occur in cells with low chromatin density (S3G Fig), which excludes apoptotic cells.

### CdtB-induced nucleoplasmic reticulum concentrates ribonucleoparticles with translational and mRNA decay activities

High levels of UNR and the cytoplasmic isoform of polyadenylate-binding protein-1 (PABPC1/PABP1) were previously reported in NR [6]. *In vivo*, these translation regulators are direct partners [19] and were found colocalized in high quantity in CdtB-induced NR core (Fig 6A). As expected, the nuclear isoform PABPN1/PABP2 (nuclear polyadenylate-binding protein-1/nuclear polyadenylate-binding protein 2) was found exclusively in the nucleus (S4 Fig).

Translation initiation in eukaryotes requires the involvement of multiple initiation factors. PABPC1 binds to PABP-interacting protein 1 (PAIP1) to enhance translation. PABPC1 also binds to eIF4G (eukaryotic translation Initiation Factor 4G), a component of the eIF4F complex containing eIF4E (eukaryotic translation Initiation Factor 4E), enhancing both the affinity of eIF4E for the cap structure and PABPC1 for poly (A), thus effectively locking proteins onto both ends of the mRNA. Immunostaining of engrafted cells (Fig 6A and 6C) showed that CdtB-induced-NRs concentrate the translational initiation factors PABPC1, PAIP1, eIF4G, eIF4E, as well as eIF4E nucleocytoplasmic shuttle protein 4E-Transporter (EIF4ENIF1 or 4E-T). Translation elongation (EEF2 for Eukaryotic Elongation Factor 2) and termination (eRF1 and eRF3, eukaryotic peptide chain Release Factor subunit 1/3) associated factors were also found in NR but these proteins did not concentrate in these structures in contrast to the translational initiation-associated proteins (S4 Fig).

Polyadenylation is crucial for mRNA stability and translation initiation. The oligo (dT) labeling revealed that mRNA were distributed throughout the cytoplasm and concentrated within UNR-NR in engrafted cells (Fig 6C and 6E), suggesting that mRNA nucleocytoplasmic transport would be favored to these structures.

A two-fold increase in ribosomes was found in the CdtB-induced NR compared to cytoplasm (RPL10, Fig 6B and 6E). Consequently, Hep3B transgenic living cells were subjected to ribopuromycilation assay to detect translation. Translating ribosomes were found to be distributed throughout the cytoplasm for all the experimental conditions and colocated with UNR in most of the CdtB-induced NR (Fig 6D and 6E). Comparative quantification of the translating ribosome proteins revealed a slight, but significant increase of translating ribosomes in UNR-NR *vs* cytoplasm. It should be noted, that during this *in vitro* assay, a soft digitonin membrane permeabilization was performed. Because NR concentrates many proteins and mRNA, it is possible that it forms an additional barrier restricting the access of puromycin to the core of the invaginations.

UNR and PABPC1 are subunits of the major coding-region determinant (mCRD)-mediated mRNA instability complex in association with 3 other RNA binding proteins, i.e. PAIP1, NSAP1 and AUF1 [20]. As for UNR, both PAIP1 and NSAP1 were found concentrated in the core of NR and colocated with PABPC1 (Fig 6A and 6E). The analysis of the last partner of mCRD-mediated complex, p37 AUF1, is complicated by the existence of four isoforms generated by alternative splicing of AUF1 transcript [21] and no antibody targeting specifically AUF1 cytosolic isoforms can be produced. p42/45 AUF1 nuclear isoforms are mainly located in the nucleus, while the p37/40 isoforms are more weakly expressed in the cytoplasm. AUF1 detection revealed a strong nuclear signal and a weak signal in cytoplasm and NR (Fig 6A and 6E). Nevertheless, the concentration of a majority of mCRD complex components and of the RNA binding protein DEAD-box helicase, DDX6 [22] (Fig 6B and 6E) in the CdtB-induced NR, suggests these structures contain many *trans*-acting regulators involved in mRNA stability [23].

Together, these data suggest CdtB-induced-NR are active sites of mRNA translation and decay.

## Discussion

Herein, we report the formation of nuclear membrane invagination/NR during bacterial infection: *H*. *hepaticus* infection triggers NR formation in the hepatocytes of infected mice. Coculture experiments with *H*. *hepaticus* and *H*. *pullorum* showed that these two Helicobacters are associated with the formation of NR. In contrast, NR formation was almost absent in cells infected with the corresponding CDT-knockout mutant strains showing that CDT is the main factor responsible for NR formation. The transgenic cell line expressing the CdtB, and especially that expressing the H265L mutated CdtB lacking catalytic activity, allowed to attribute NR formation to the CdtB subunit. NR formation was associated with a profound nuclear remodeling following *H*. *hepaticus* infection and a significant increase in nucleus size. Moreover, the bigger the nuclei, the stronger the DNA damage. Colibactin, another bacterial genotoxin, also triggered NR formation, suggesting that NR formation is a consequence of genotoxic-induced DNA damage. This hypothesis is supported by the fact that etoposide, a potent DNA damaging agent, also induces NR formation [5]. The tight association of NR with γH2AX-positive DNA lesions following γ-radiation and the regulator of cellular response to DNA damage 53BP1 [24] constitute an additional argument to support a role for a dynamic NR formation in DNA damage repair.

NR have not been previously reported during bacterial infection in NR-susceptible cell lines (Vero, Hela and CHO), even for bacteria whose toxin is targeted to the ER [7]. Unlike other ER-translocating toxins acting in the cytosol, CdtB is subjected to an atypical translocation mechanism from the ER to the nucleus, a nuclear access *via* NR would facilitate CdtB transport to the nucleus where the toxin damages host-cell DNA. GST-tagged CdtB and ER were reported once previously to be colocated at NR-like structure in HeLa cells but this NR was pre-existing in uninfected cells, suggesting it was a normal cellular structure rather than a byproduct of CdtB intoxication [25]. Immunostaining of transgenic intestinal SW480 cells expressing the CdtB of *H*. *hepaticus* showed that NR did not concentrate CdtB (S5A Fig). It is thus unlikely that NR sustain the nucleo-cytoplasmic shuttling of the CdtB to the nucleus where the toxin exerts its toxicity. In addition, quantification of CdtB in NR- versus NR+ cells did not reveal significant differences between the levels of CdtB expression and NR formation (S5B Fig).

Virus and protozoa infections also induce jagged appearance of the nuclear lamina with evidence of invaginations of the nuclear envelope [7], but presenting a different composition compared to CdtB-induced NR. Indeed, the cytomegalovirus and alphaherpesvirus-induced NR are devoid of ribosomes and mitochondria [26] and *Toxoplasma gondii*-induced NR (parasitophorous vacuole) show little specific colocalization with markers for host ER or mitochondria [27]. Thus, CdtB-induced UNR-NR are structures different to those observed in response to virus and protozoa infections. This, again, emphasizes the unique aspects of CDT intoxication.

Many cytoplasmic proteins are present in CdtB-induced NR but at a level similar to that in the cytoplasm, probably randomly included during NR formation. The composition of CdtB-induced NR is reminiscent of that of ribonucleoprotein granules, such as stress granules (SG), processing bodies (GW/P-bodies), three structurally and dynamically linked compartments important in the post-transcriptional regulation of gene expression [28]. CdtB-induced NR shares some proteins and mRNA components (eIF4E, DDX6) either with SG and GW/P-bodies but ultrastructure and protein composition of these NR revealed divergences to that of SG and GW/P-bodies [29]. It is unlikely that CdtB-induced NR correspond to SG or GW-/P-bodies invaginated in the nucleus, since these bodies were never detected in the cytoplasm of the CdtB intoxicated cells. The accumulation of the PABPC1 and eIF4G in CdtB-induced NR, two markers selectively recruited to SG but missing in GW/P-bodies, as well as the absence of accumulation of the GW/P-bodies proteins (GW182 and AGO2), allow to definitively exclude that CdtB-induced NR correspond to GW/P-bodies invaginated in the nucleus. Besides, the size of these NR (in the range of μm) would be similar to that observed in SG [30]. However, the non-spheroid/ellipsoid structures of CdtB-induced NR, the concentration of the large 60S ribosomal subunits RPL10 excluded in SG [31], and the lack of accumulation of TIA1 (T cell intracellular antigen), a common SG marker, do not allow to conclude that CdtB-induced NR are SG invaginated into the nucleus. Additionally, SG are distant from the nuclear envelope, mitochondria and ER [29] while CdtB-induced NR are deeply invaginated into nuclei and tightly linked to these organelles.

CdtB and colibactin triggered the formation of messenger ribonucleoprotein (mRNP) particles clustered and invaginated into deep nuclear channels. Compared to the cytoplasm, a slight increase of translation was observed in these dynamic particles, whose formation is dependent on an active translation. These transient particles, induced in response to DNA damage, also concentrate and insulate mRNAs, preinitiation factors and specific RNA-binding proteins, such as the subunits of the mCRD complex involved in mRNA destabilization [20]. Accordingly, CdtB-induced NR may be transient signaling hubs controlling mRNA turnover and translation of selected mRNAs. Indeed, already formed NR invaginations were shown to be resorbed back into the envelope, supporting the idea that NR events are reversible and dynamic. [32]

Silencing experiments revealed that 1) UNR is essential for the survival of CdtB-expressing cells *in vitro* and 2) NRs resist to RNA interference-mediated UNR protein depletion and is implicated in cell survival, which is in line with an active mRNA translation and decay in these structures. During UNR silencing, the absence of extinction of UNR signal only in NR in CdtB-Hep3B giant surviving cells may be explained by various compatible hypotheses. CdtB-NR are dense particles formed by long narrow tubular channels ending with a core concentrating mRNA and RNA binding proteins, likely resulting in a restricted access of the UNR siRNA to the nuclear invaginations. As proteins are concentrated in NR, their stability would also be favored. Moreover, NRs are site of mRNA accumulation, suggesting that nucleo-cytoplasmic transport of mRNA *via* the nuclear pores might be preferentially directed from the nucleus to these structures deeply invaginated in the nucleoplasm rather than to the cytoplasm. CdtB-induced NR would thus be a privileged gateway for selected mRNA, as UNR mRNA, preferentially transported therein for local translation in this protected structure, and thus able to avoid the cytoplasmic siRNA. The presence of a cytoplasmic core and NPC around NR supports a possible role for these structures in nucleo-cytoplasmic transport. Clear association of a subset of NR with nucleoli would also be consistent with a role in nuclear RNA export [33]. Additionally, numerous CdtB-induced NR contained cytoskeletal elements that are thought to facilitate trafficking of mRNAs and organites such as mitochondria [34]. Thus, UNR-NR would be essential dynamic cellular structures involved in the survival of DNA-damaged cells. UNR-NR would be the hubs that intercept a subset of signaling molecules, thereby communicating a “state of emergency” to other signaling pathways, modulating metabolism, growth and survival.

CdtB-induced NR is surrounded by the nuclear lamina tightly linked with chromatin, suggesting a role for chromatin in NR formation. The fact that condensins exert force on chromatin-nuclear envelope tethers to mediate NR formation [32], supports this hypothesis in CdtB-induced NR.

UNR-NR was also present at a basal level in the hepatocytes of non-infected mice and in cancer derived-cell lines (Hep3B and SW480). *In vivo* Unr expression study in mice using tissue microarray showed that the formation of UNR-NR was restricted to trophoblast giant cells in the developing placenta and hepatocytes [6], the two polyploid cell types resulting from endoreplication. Endoreplication is also known to confer genome instability [35]. As UNR-NR formation is associated with DNA damage/repair, the formation of UNR-NR at a basal level may be a cellular response to genome instability triggered by endoreplication. Genomic instability that accumulates in cancer derived-cell lines (Hep3B and SW480) may also lead to UNR-NR formation in those cells.

CdtB promotes endoreplication leading to giant polyploid cells,[10] these latter cells presented the highest and largest UNR-NR. Since endoreplication and polyploidy are required for the maintenance of cell identity, CdtB-induced NR may occur in giant endoreplicating polyploid cells that would rewire DNA damage response networks to overcome replication stress-induced barriers, giving advantages for cell survival and growth. This is supported by the fact that NR can be formed *de novo* without mitosis [36] and tumor cells can evade DNA-damage through DNA endoreduplication and reversible polyploidy. [37]

While senescence and apoptosis were observed in our models (coculture experiments and xenograft mice), we found no association between CdtB-induced NR formation and apoptosis or senescence phenotypes, constituting an additional argument to support a role for NR in cell survival. Indeed, cellular senescence is often considered as a terminal cell state, but it was shown to be reversible in some polyploid cancer cells following DNA damage. [38] Overcoming the genotoxic damage in those cancer cells is associated with reversible polyploidy which coincides with reversible senescence [37]. Here, the CdtB-intoxicated giant polyploid cells accumulate NR, survive and keep proliferating with an apparent NR resorption and return back to normal size, suggesting de-polyploidisation. Thus, we can assume that those CdtB-intoxicated polyploid cells might have entered cellular senescence and rewired the DNA damage response and repair networks to escape the genotoxic stress-associated senescence,[39] leading to cell survival and de-polyploidisation. Additionally, the presence of calnexin in NR may maintain a Ca^2+^ store in close proximity to the nuclear matrix, allowing the transport of Ca^2+^ to the nucleus, which is necessary to regulate gene transcription as well as cell proliferation.

The CdtB-induced invaginations were less notable in the presence of inhibitors targeting the MAPK/ERK, JNK and p38 MAPK pathways, as well as with SERCA Ca^2+^ channel blockers. Ca^2+^ regulates ERK signaling [40]. Knowing that elevated Ca^2+^ concentration [41] and CDT activates the MAPK/ERK cascade,[42] SERCA and ERK/MAPK inhibitors would counteract the CDT effects and therefore reduced the number of CdtB-induced NR. NR is known to facilitate Ca^2+^ exchanges between the cytoplasm and the nucleoplasm, and to preserve the ability to generate nucleoplasmic Ca^2+^ transients in cells *via* IP3R and ryanodine receptors [18]. CdtB-induced NR probably sustains increased expression of genes regulated by localized Ca^2+^ release and increases export of mRNA for translation (NR and Ca^2+^ signaling reviewed in [7]).

CDT intoxication was associated with various phenotypes, *i*.*e*. apoptosis and cell death, DNA proper or improper repair and cell survival, senescence and endoreduplication. Here, we showed that the genotoxic stress induced by bacterial genotoxin can also promote the formation of NR deeply invaginated in the nucleoplasm of giant nuclei together with profound nuclear reorganization (chromatin remodeling, hyperploidization). The core of the genotoxin-induced NR concentrates proteins involved in mRNA translation, polyadenylated RNA, ribosomes, as well as the main subunits of the mCRD complex involved in mRNA turnover. These dynamic cellular structures are active sites of mRNA translation and decay. Their formation has not been previously reported to occur during bacterial infections. They may correspond to a privileged gateway for the synthesis of selected mRNA preferentially transported from the nucleus through pores and translated therein. Our conclusion is that these transient reversible structures allow the cell to pause and repair the DNA damage caused by bacterial genotoxins to maintain cell survival (S6 Fig). In this latter case, NR may contribute to the resistance of cancer cells to radiotherapies and some chemotherapies inducing DNA damage.

## Supporting information

S1 Fig *In vivo* detection of UNR protein in liver of mice infected with *Helicobacter hepaticus*. Images of mouse livers following a 12 months infection with *H*. *hepaticus*. Widefield image of 3 μm-tissue sections tissue sections of mice liver stained with fluorescent primary and secondary antibodies targeting UNR (green) and DAPI to counterstain the nucleus (blue). White arrowheads indicate UNR-NR. Tumoral areas are delineated by a discontinuous line. Enlargement of immunofluorescent staining are shown in boxes. DAPI, 4′, 6′-diamidino-2-phenylindol.
(PDF)

S2 Fig Detection of UNR protein in mice stomachs during *Helicobacter felis* infection. C57BL/6J mice were infected with *H*. *felis* strain CS1 (n = 5) for 55 weeks [15]. Non-infected mice (Brucella broth, n = 5) were used as concurrent controls. Three μm-tissue sections of paraffin embedded gastric specimens were subjected to standard hematoxylin staining and immunostaining raised against UNR. Enlargement of immunohistochemical staining are shown in boxes. Representative images of the non-infected stomach **(A)** or stomach infected with *H*. *felis* presenting metaplasia **(B)** and dysplasia **(C)** are shown. Green, yellow and pink arrows indicate pseudointestinal metaplasia, mucinous metaplasia and dysplasia, respectively.
(PDF)

S3 Fig *In vitro* detection of UNR-NR during bacterial infection. Liver Hep3B **(A)** and colon SW480 **(B)** cells were infected for 72 h with *H*. *pullorum* strain H495, its corresponding CDT-knockout mutant strain (ΔCDT), or with *H*. *pylori* strain 7.13 at a multiplicity of infection (MOI) of 100 bacteria/cell and cells were maintained for 72 hours prior analysis. A coculture with *E*. *coli* strain secreting the Shiga toxin-2 was also conducted for 6 h at a MOI of 100 bacteria/cell and cells were maintained for 72 hours prior analysis. **(C)** Gastric AGS cells were infected for 6 h with *E*. *coli* strain harboring the pks genomic island encoding colibactin (BAC pks) and the corresponding bacterial artificial chromosome (BAC), *E*. *coli* strain secreting the Shiga toxin-2, as well as with *H*. *pylori* strain 7.13 at a MOI of 100 bacteria/cell and cells were maintained for 72 hours prior analysis. **D)** Concurrently, AGS cells were infected for 24 h with *H*. *pylori* strain 7.13 at a MOI of 25 bacteria/cell to verify the “hummingbird” phenotype. Non-infected cells were used as controls in all experiments. Cells were stained with fluorescent primary and secondary antibodies targeting UNR (green), DAPI to counterstain the nucleus (blue) and fluorescent-labeled phalloidin to detect F-actin (red, only in D). Yellow, blue and white arrowheads indicate UNR-NR, cells presenting a hummingbird-like phenotype and cells undergoing mitosis, respectively. Fluorescent staining was observed using widefield fluorescence imaging as previously reported [44]. **(E)** Hep3B transgenic cells were cultivated with doxycycline for 72 h to induce the expression of the control Red Fluorescent Protein (RFP), the CdtB of *H*. *hepaticus* strain 3B1 (CdtB) or the CdtB of *H*. *hepaticus* strain 3B1 with the H265L mutation (CdtB-H265). Cells were then processed for Western blot analysis with antibodies generated against UNR (1/1000, HPA018846, Sigma) and α-tubulin (1/5000, T9026, Sigma), this latter protein was used as a reference protein [44]. Each membrane was used for both proteins detection. Subsequent quantifications were performed with ImageJ (v. 1.52n) [54] using capture of staining, each count being performed on 4 analyses. The level of UNR expression was normalized to tubulin prior comparison between the 3 conditions. The discontinuous line shows the basal rate of UNR expression by RFP cells. **(F)** Quantification of the γH2AX signal in Hep3B transgenic cells cultivated as in Fig 3C. Cells were stained with fluorescent primary and secondary antibodies targeting γH2AX (green) and UNR (red), and DAPI to counterstain the nucleus (blue). γH2AX foci-positive nuclei were classified according to the average volume of the nuclei calculated in the cells expressing the RFP: 194.40 μm^3^. At least 200 cells were counted for each experiment. Data represent the mean of triplicates in 1 representative experiment of 3. **(G)** Quantification of the DAPI staining in the nucleoplasm of CdtB-expressing Hep3B engrafted cells from Fig 3C was performed with ImageJ (v. 1.52n) [54] using capture of fluorescent staining (confocal imaging), each count being performed on 100 nuclei. **(H)** Hep3B transgenic cells were cultivated as in Fig 3C. Then, cells were submitted to β-galactosidase and fluorescent staining using the same slide. First, the Senescence β-Galactosidase Staining kit (Cell Signaling) was used according to the supplier's recommendations. Second, cells were stained with fluorescent primary and secondary antibodies targeting UNR (green), and DAPI to counterstain the nucleus (blue). Imaging combining the β-galactosidase signal detection and the detection of fluorescent signals was obtained using successively transmitted light and fluorescence microscopy (Zeiss Axioplan 2 fluorescence microscope, Zeiss, Jena, Germany). β-galactosidase signal was converted in artificial red and merged with the immunofluorescent signals using ImageJ (v. 1.52n) [54]. *** p<0.001 AU, arbitrary units; Tub, tubulin; ns, not significant.
(PDF)

S4 Fig Subcellular localization of proteins in response to the CdtB of *Helicobacter hepaticus*. Confocal imaging of Hep3B CdtB-expressing cells engrafted in mice (3 μm-tissue section) or transgenic CdtB-expressing cells* cultivated with doxycycline for 72 h was performed (as in Figs 3A and 6A). Tissues/cells were processed for fluorescent staining with primary antibodies (associated with fluorescent-labeled secondary antibodies) generated against the proteins of interest (green) and NPC, calnexin, UNR or PABPC1 (red), depending on the origin of an antibody used to detect the protein of interest. Subsequent quantification of the proteins in nucleoplasm, cytoplasm and foci were performed using capture of fluorescent staining (confocal imaging) by measuring the pixel intensity with the “Plot Profile” function of ImageJ (v. 1.52n) [54], each count being performed on 100 NRs. The relative expression rate of protein in NR in response to the CdtB was reported as a fold increases versus the expression in the cytosol, with the exception of PABPN1 and GW182 absent in NR and calnexin due to lamellar staining. The discontinuous line shows the basal rate in the cytoplasm. AGO2, Protein argonaute-2; C, cytoplasm; EEF2, eukaryotic elongation factor 2; eRF, eukaryotic release factors; GW182, trinucleotide repeat-containing gene 6A protein; IP3R2 and IP3R3, Inositol 1,4,5-trisphosphate receptor type 2 and 3; N, nucleus; NPC, Nuclear Pore Complex; PABPC1, cytoplasmic isoform of polyadenylate-binding protein 1; PABPN1, nuclear isoform of polyadenylate-binding protein 1; TIA1, T cell intracellular antigen; UNR, upstream of N-RAS. *Transgenic CdtB-expressing cells and ethanol permeabilization.
(PDF)

S5 Fig Subcellular localization of proteins in response to the CdtB of *Helicobacter hepaticus* in intestinal cells. Imaging of SW480 intestinal cells expressing the CdtB of *H*. *hepaticus* fused at its 3′ end to three repeats of the influenza hemagglutinin epitope (HA). Cells were processed for fluorescent staining with primary antibodies (associated with fluorescent-labeled secondary antibodies) generated against UNR (red) and the HA tag of the CdtB (red) as well as with DAPI to counterstain the nucleus (blue). Widefield and confocal imaging showed that CdtB did not colocalize with NR. **(A)** Widefield imaging showed that CdtB was detected mainly in the nucleus and excluded from the nucleoli (pink arrowheads), as expected [44]. **(B)** Confocal imaging showed that CdtB was detected in the cytoplasm, nucleus and excluded from the nucleoli. CdtB was also detected at the cell periphery lamellipodia and membrane ruffles (green arrows), as expected [44]. Subsequent quantification of the 3HA-tagged CdtB was performed using capture of fluorescent staining (confocal imaging) by measuring the pixel intensity with the “Plot Profile” function of ImageJ 1.51 [54], each count was performed on 100 cells. Yellow and pink arrowheads indicate UNR-NR and nucleoli, respectively. NR, nucleoplasmic reticulum. ns, not significant.
(PDF)

S6 Fig Cytolethal distending toxin induces the formation of transient messenger-rich ribonucleoprotein nuclear invaginations in surviving cells 1- Bacterial genotoxin infection and cytoplasmic CdtB active subunit internalization 2- Giant nuclei together with profound nuclear reorganization in response to DNA damage—3- Cell cycle pause and DNA damage repair 4- Cell survival and cell cycle re-entry.
(PDF)

S1 Table Antibodies used for immunohistochemistry and immunocytochemistry experiments. (DOCX)

S1 Methods (PDF)

S1 Results (PDF)
