# Supplementary material for: Cytolethal distending toxin induces the formation of transient messenger-rich ribonucleoprotein nuclear invaginations in surviving cells
Source: PLoS Pathog. 2019 Sep 30;15(9):e1007921. doi: 10.1371/journal.ppat.1007921 (PMC6824578; doi:10.1371/journal.ppat.1007921)
Supplement: S1 Fig — Images of mouse livers following a 12 months infection with H. hepaticus. Widefield image of 3 μm-tissue sections tissue sections of mice liver stained with fluorescent primary and secondary antibodies targeting UNR (green) and DAPI to counterstain the nucleus (blue). White arrowheads indicate UNR-NR. Tumoral areas are delineated by a discontinuous line. Enlargement of immunofluorescent staining are shown in boxes. DAPI, 4′, 6′-diamidino-2-phenylindol. (PDF) [file ppat.1007921.s001.pdf]

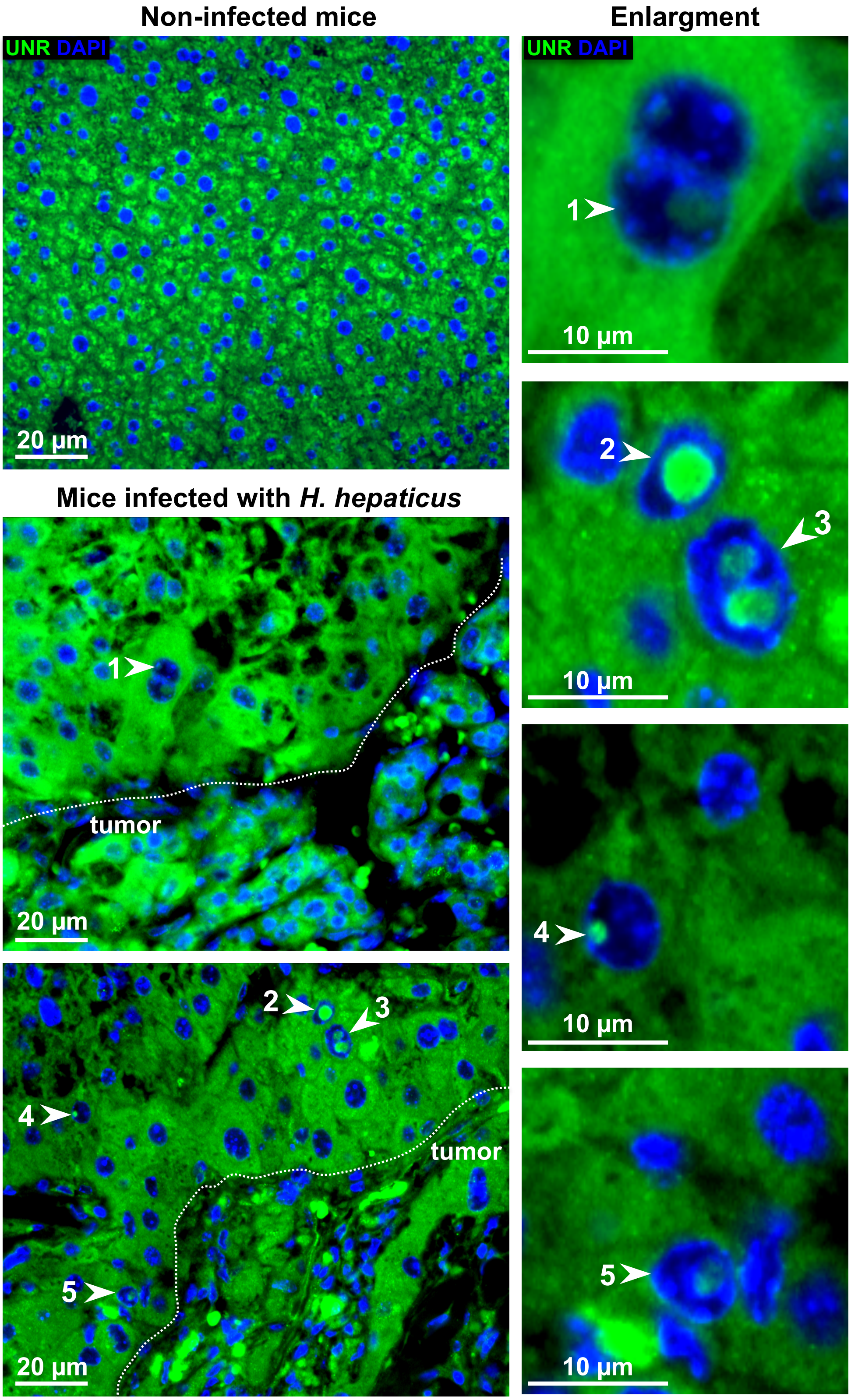

S1 Fig. *In vivo* detection of UNR protein in liver of mice infected with *Helicobacter hepaticus*. Images of mouse livers following a 12 months infection with *H. hepaticus*. Widefield image of 3 µm-tissue sections tissue sections of mice liver stained with fluorescent primary and secondary antibodies targeting UNR (green) and DAPI to counterstain the nucleus (blue).
White arrowheads indicate UNR-NR. Tumoral areas are delineated by a discontinuous line.
Enlargement of immunofluorescent staining are shown in boxes.
DAPI, 4′, 6′-diamidino-2-phenylindol.
