# Supplementary material for: Cytolethal distending toxin induces the formation of transient messenger-rich ribonucleoprotein nuclear invaginations in surviving cells
Source: PLoS Pathog. 2019 Sep 30;15(9):e1007921. doi: 10.1371/journal.ppat.1007921 (PMC6824578; doi:10.1371/journal.ppat.1007921)
Supplement: S2 Fig — C57BL/6J mice were infected with H. felis strain CS1 (n = 5) for 55 weeks [15]. Non-infected mice (Brucella broth, n = 5) were used as concurrent controls. Three μm-tissue sections of paraffin embedded gastric specimens were subjected to standard hematoxylin staining and immunostaining raised against UNR. Enlargement of immunohistochemical staining are shown in boxes. Representative images of the non-infected stomach (A) or stomach infected with H. felis presenting metaplasia (B) and dysplasia (C) are shown. Green, yellow and pink arrows indicate pseudointestinal metaplasia, mucinous metaplasia and dysplasia, respectively. (PDF) [file ppat.1007921.s002.pdf]

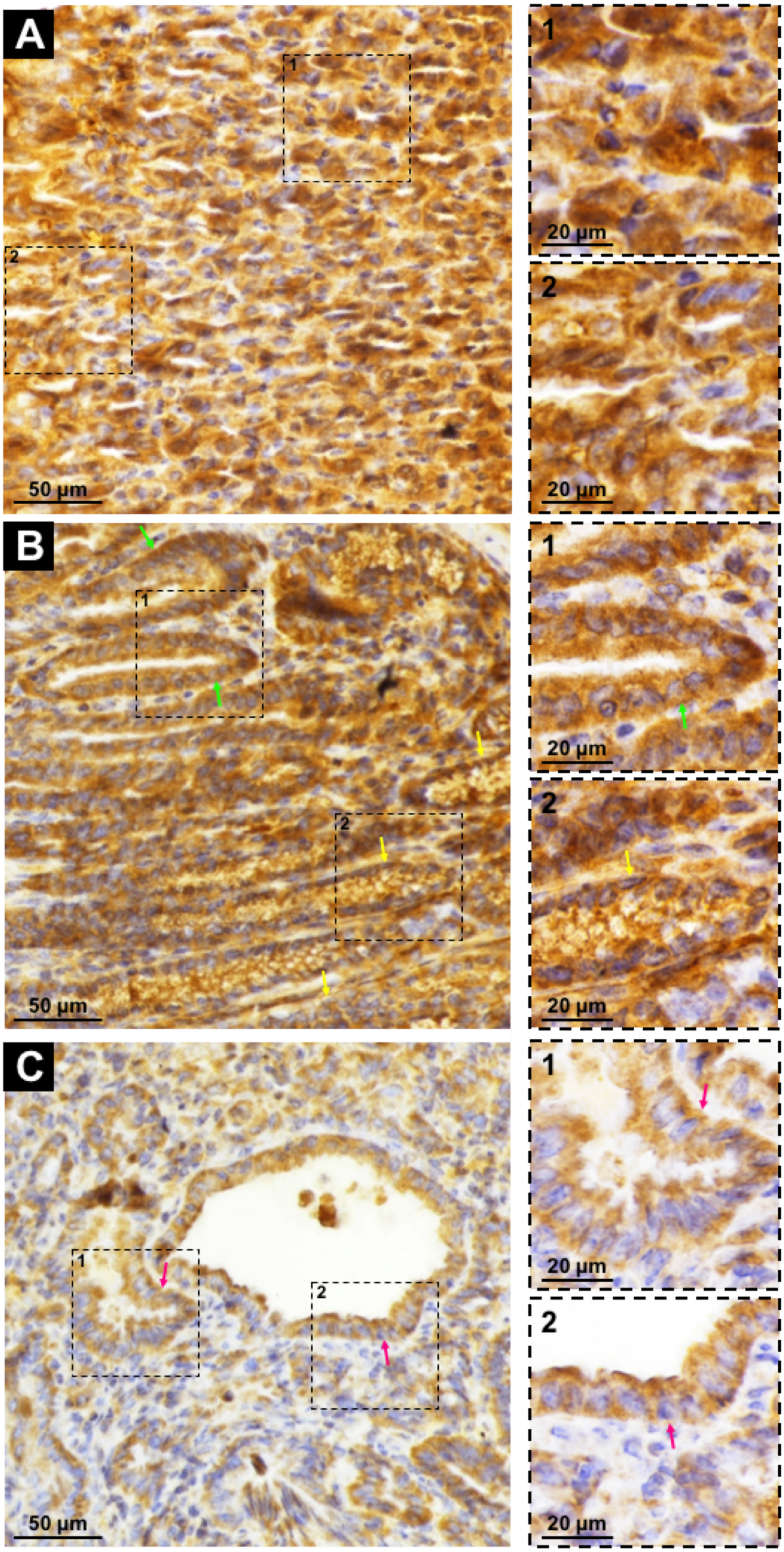

S2 Fig. Detection of UNR protein in mice stomachs during *Helicobacter felis* infection.
C57BL/6J mice were infected with *H. felis* strain CS1 (n=5) for 55 weeks [15]. Non-infected mice (Brucella broth, n=5) were used as concurrent controls. Three µm-tissue sections of paraffin embedded gastric specimens were subjected to standard hematoxylin staining and immunostaining raised against UNR. Enlargement of immunohistochemical staining are shown in boxes.
Representative images of the non-infected stomach **(A)** or stomach infected with *H. felis* presenting metaplasia **(B)** and dysplasia **(C)** are shown. Green, yellow and pink arrows indicate pseudointestinal metaplasia, mucinous metaplasia and dysplasia, respectively.
