# Supplementary material for: Cytolethal distending toxin induces the formation of transient messenger-rich ribonucleoprotein nuclear invaginations in surviving cells
Source: PLoS Pathog. 2019 Sep 30;15(9):e1007921. doi: 10.1371/journal.ppat.1007921 (PMC6824578; doi:10.1371/journal.ppat.1007921)
Supplement: S3 Fig — Liver Hep3B (A) and colon SW480 (B) cells were infected for 72 h with H. pullorum strain H495, its corresponding CDT-knockout mutant strain (ΔCDT), or with H. pylori strain 7.13 at a multiplicity of infection (MOI) of 100 bacteria/cell and cells were maintained for 72 hours prior analysis. A coculture with E. coli strain secreting the Shiga toxin-2 was also conducted for 6 h at a MOI of 100 bacteria/cell and cells were maintained for 72 hours prior analysis. (C) Gastric AGS cells were infected for 6 h with E. coli strain harboring the pks genomic island encoding colibactin (BAC pks) and the corresponding bacterial artificial chromosome (BAC), E. coli strain secreting the Shiga toxin-2, as well as with H. pylori strain 7.13 at a MOI of 100 bacteria/cell and cells were maintained for 72 hours prior analysis. D) Concurrently, AGS cells were infected for 24 h with H. pylori strain 7.13 at a MOI of 25 bacteria/cell to verify the “hummingbird” phenotype. Non-infected cells were used as controls in all experiments. Cells were stained with fluorescent primary and secondary antibodies targeting UNR (green), DAPI to counterstain the nucleus (blue) and fluorescent-labeled phalloidin to detect F-actin (red, only in D). Yellow, blue and white arrowheads indicate UNR-NR, cells presenting a hummingbird-like phenotype and cells undergoing mitosis, respectively. Fluorescent staining was observed using widefield fluorescence imaging as previously reported [44]. (E) Hep3B transgenic cells were cultivated with doxycycline for 72 h to induce the expression of the control Red Fluorescent Protein (RFP), the CdtB of H. hepaticus strain 3B1 (CdtB) or the CdtB of H. hepaticus strain 3B1 with the H265L mutation (CdtB-H265). Cells were then processed for Western blot analysis with antibodies generated against UNR (1/1000, HPA018846, Sigma) and α-tubulin (1/5000, T9026, Sigma), this latter protein was used as a reference protein [44]. Each membrane was used for both proteins detection. Subseque [file ppat.1007921.s003.pdf]

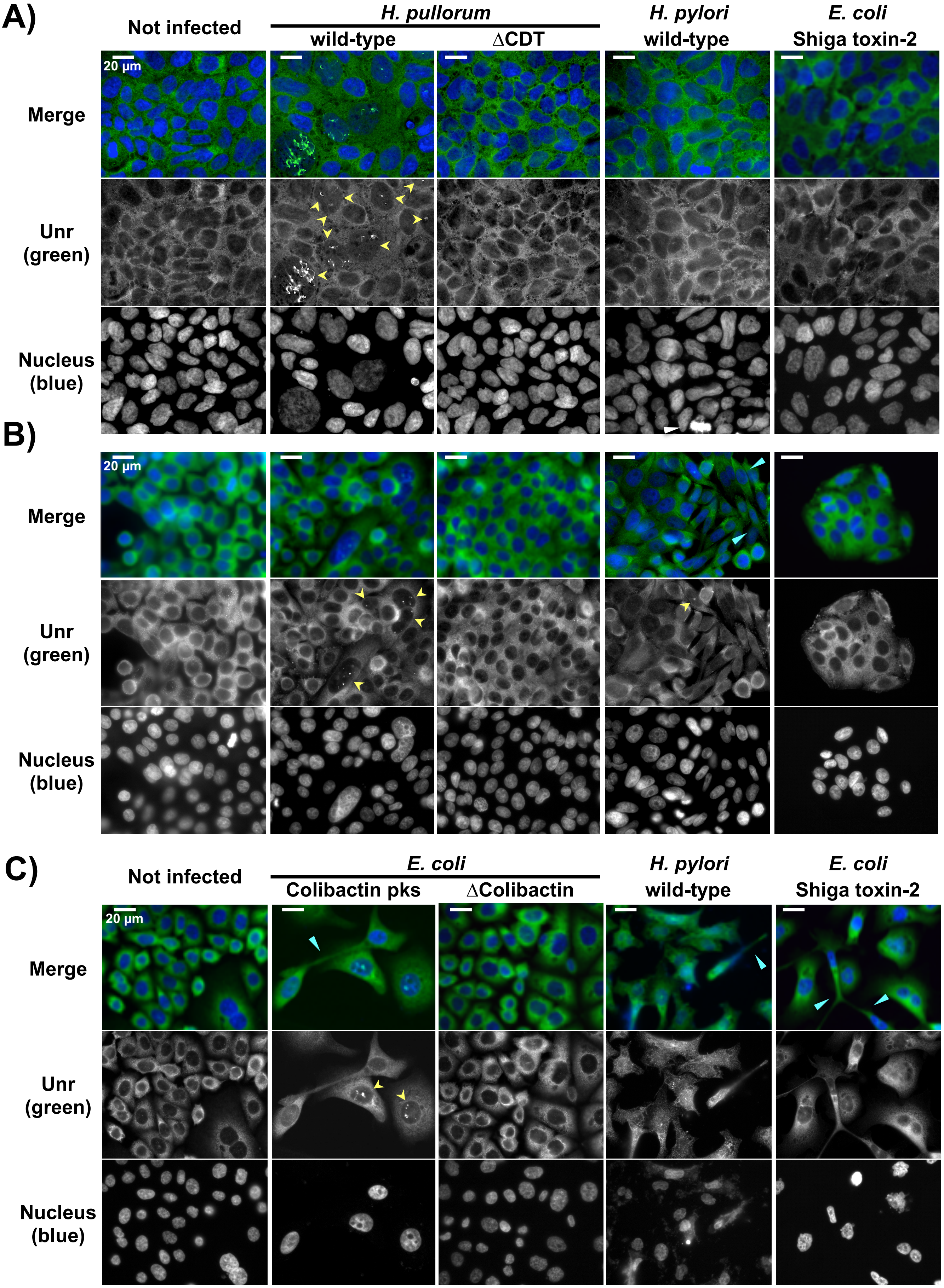


**S3 Fig.** ***In vitro*** **detection of UNR-NR during bacterial infection.**

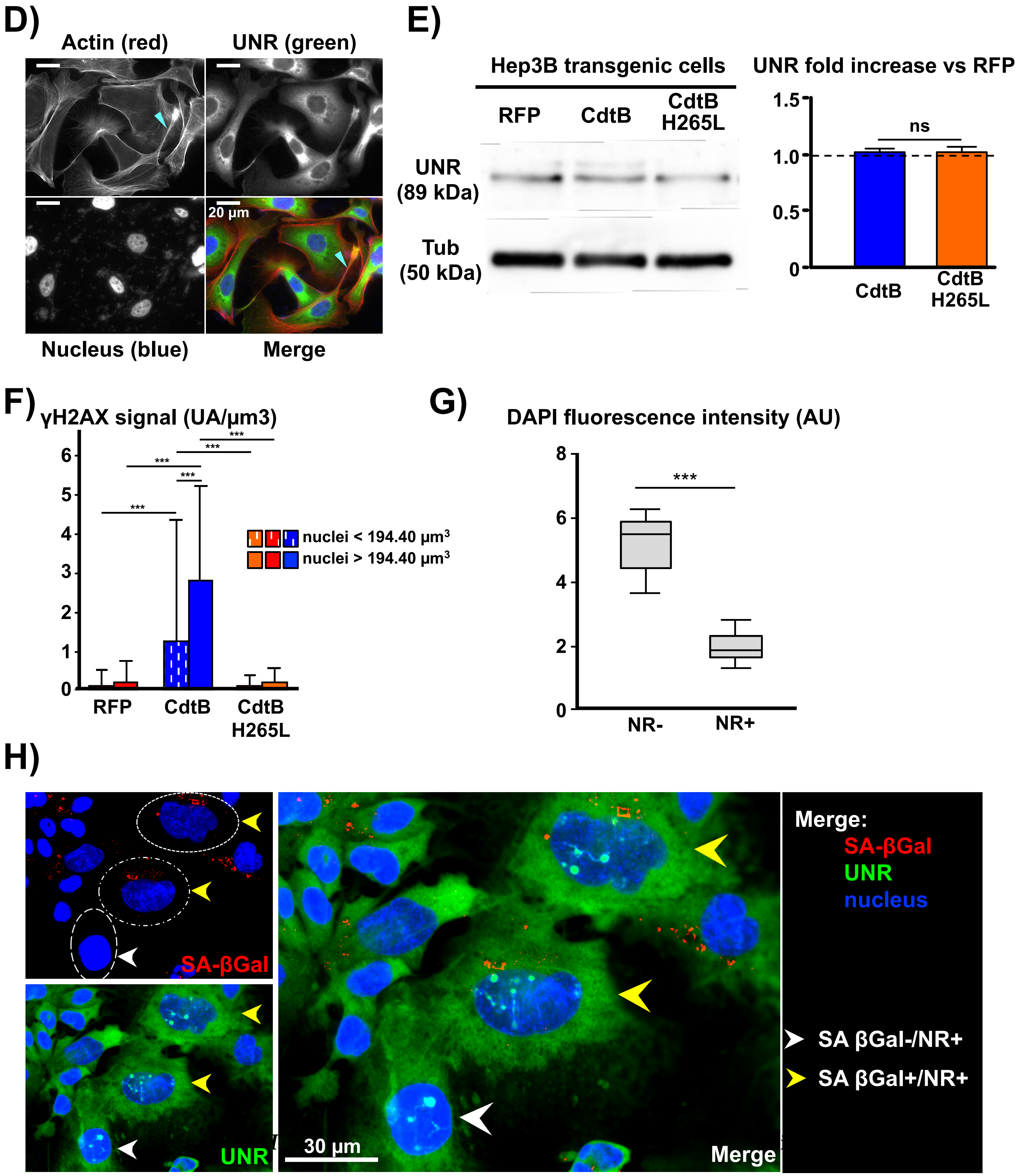
D)
Actin (red)
UNR (green)
20 µm
Nucleus (blue)
Merge
E)
Hep3B transgenic cells
RFP
CdtB
CdtB H265L
UNR (89 kDa)
Tub (50 kDa)
UNR fold increase vs RFP
1.5
1.0
0.5
0
ns
CdtB
CdtB H265L
F)
γH2AX signal (UA/µm3)
6
5
4
3
2
1
0
***
nuclei < 194.40 µm3
nuclei > 194.40 µm3
RFP
CdtB
CdtB H265L
G)
DAPI fluorescence intensity (AU)
8
6
4
2
0
***
NR-
NR+
H)
SA-βGal
UNR
30 µm
Merge
Merge:
SA-βGal
UNR
nucleus
SA βGal-/NR+
SA βGal+/NR+

**S3 Fig. *In vitro* detection of UNR-NR during bacterial infection.**

Liver Hep3B **(A)** and colon SW480 **(B)** cells were infected for 72 h with *H. pullorum* strain H495, its corresponding CDT-knockout mutant strain (ΔCDT), or with *H. pylori* strain 7.13 at a multiplicity of infection (MOI) of 100 bacteria/cell and cells were maintained for 72 hours prior analysis. A coculture with *E. coli* strain secreting the Shiga toxin-2 was also conducted for 6 h at a MOI of 100 bacteria/cell and cells were maintained for 72 hours prior analysis. **(C)** Gastric AGS cells were infected for 6 h with *E. coli* strain harboring the pks genomic island encoding colibactin (BAC pks) and the corresponding bacterial artificial chromosome (BAC), *E. coli* strain secreting the Shiga toxin-2, as well as with *H. pylori* strain 7.13 at a MOI of 100 bacteria/cell and cells were maintained for 72 hours prior analysis. **D)** Concurrently, AGS cells were infected for 24 h with *H. pylori* strain 7.13 at a MOI of 25 bacteria/cell to verify the "hummingbird" phenotype. Non-infected cells were used as controls in all experiments. Cells were stained with fluorescent primary and secondary antibodies targeting UNR (green), DAPI to counterstain the nucleus (blue) and fluorescent-labeled phalloidin to detect F-actin (red, only in D). Yellow, blue and white arrowheads indicate UNR-NR, cells presenting a hummingbird-like phenotype and cells undergoing mitosis, respectively. Fluorescent staining was observed using widefield fluorescence imaging as previously reported [44]. **(E)** Hep3B transgenic cells were cultivated with doxycycline for 72 h to induce the expression of the control Red Fluorescent Protein (RFP), the CdtB of *H. hepaticus* strain 3B1 (CdtB) or the CdtB of *H. hepaticus* strain 3B1 with the H265L mutation (CdtB-H265). Cells were then processed for Western blot analysis with antibodies generated against UNR (1/1000, HPA018846, Sigma) and α-tubulin (1/5000, T9026, Sigma), this latter protein was used as a reference protein [44]. Each membrane was used for both proteins detection. Subsequent quantifications were performed with ImageJ (v. 1.52n) [54] using capture of staining, each count being performed on 4 analyses. The level of UNR expression was normalized to tubulin prior comparison between the 3 conditions. The discontinuous line shows the basal rate of UNR expression by RFP cells. **(F)** Quantification of the γH2AX signal in Hep3B transgenic cells cultivated as in Fig. 3C. Cells were stained with fluorescent primary and secondary antibodies targeting γH2AX (green) and UNR (red), and DAPI to counterstain the nucleus (blue). γH2AX foci-positive nuclei were classified according to the average volume of the nuclei calculated in the cells expressing the RFP: 194.40 $\mu m^3$. At least 200 cells were counted for each experiment. Data represent the mean of triplicates in 1 representative experiment of 3. **(G)** Quantification of the DAPI staining in the nucleoplasm of CdtB-expressing Hep3B engrafted cells from Fig. 3C was performed with ImageJ (v. 1.52n) [54] using capture of fluorescent staining (confocal imaging), each count being performed on 100 nuclei. **(H)** Hep3B transgenic cells were cultivated as in Fig. 3C. Then, cells were submitted to β-galactosidase and fluorescent staining using the same slide. First, the Senescence β-Galactosidase Staining kit (Cell Signaling) was used according to the supplier's recommendations. Second, cells were stained with fluorescent primary and secondary antibodies targeting UNR (green), and DAPI to counterstain the nucleus (blue). Imaging combining the β-galactosidase signal detection and the detection of fluorescent signals was obtained using successively transmitted light and fluorescence microscopy (Zeiss Axioplan 2 fluorescence microscope, Zeiss, Jena, Germany). β-galactosidase signal was converted in artificial red and merged with the immunofluorescent signals using ImageJ (v. 1.52n) [54].

*** $p<0.001$

AU, arbitrary units;

Tub, tubulin;

ns, not significant.
