# Supplementary material for: Cytolethal distending toxin induces the formation of transient messenger-rich ribonucleoprotein nuclear invaginations in surviving cells
Source: PLoS Pathog. 2019 Sep 30;15(9):e1007921. doi: 10.1371/journal.ppat.1007921 (PMC6824578; doi:10.1371/journal.ppat.1007921)
Supplement: S4 Fig — Confocal imaging of Hep3B CdtB-expressing cells engrafted in mice (3 μm-tissue section) or transgenic CdtB-expressing cells* cultivated with doxycycline for 72 h was performed (as in Figs 3A and 6A). Tissues/cells were processed for fluorescent staining with primary antibodies (associated with fluorescent-labeled secondary antibodies) generated against the proteins of interest (green) and NPC, calnexin, UNR or PABPC1 (red), depending on the origin of an antibody used to detect the protein of interest. Subsequent quantification of the proteins in nucleoplasm, cytoplasm and foci were performed using capture of fluorescent staining (confocal imaging) by measuring the pixel intensity with the “Plot Profile” function of ImageJ (v. 1.52n) [54], each count being performed on 100 NRs. The relative expression rate of protein in NR in response to the CdtB was reported as a fold increases versus the expression in the cytosol, with the exception of PABPN1 and GW182 absent in NR and calnexin due to lamellar staining. The discontinuous line shows the basal rate in the cytoplasm. AGO2, Protein argonaute-2; C, cytoplasm; EEF2, eukaryotic elongation factor 2; eRF, eukaryotic release factors; GW182, trinucleotide repeat-containing gene 6A protein; IP3R2 and IP3R3, Inositol 1,4,5-trisphosphate receptor type 2 and 3; N, nucleus; NPC, Nuclear Pore Complex; PABPC1, cytoplasmic isoform of polyadenylate-binding protein 1; PABPN1, nuclear isoform of polyadenylate-binding protein 1; TIA1, T cell intracellular antigen; UNR, upstream of N-RAS. *Transgenic CdtB-expressing cells and ethanol permeabilization. (PDF) [file ppat.1007921.s004.pdf]

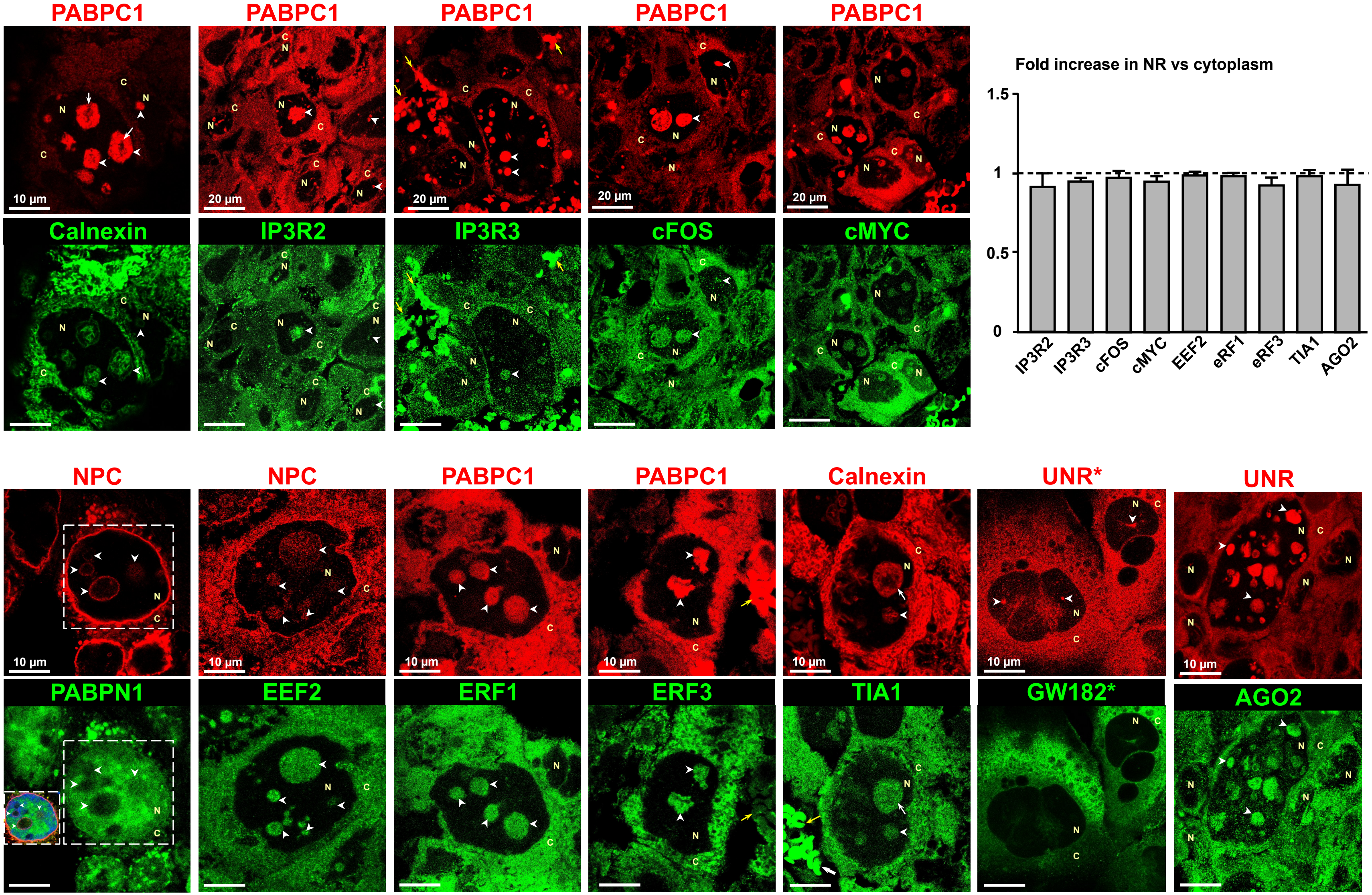
PABPC1
PABPC1
PABPC1
PABPC1
PABPC1
Calnexin
IP3R2
IP3R3
cFOS
cMYC
Fold increase in NR vs cytoplasm
1.5
1
0.5
0
IP3R2
IP3R3
cFOS
cMYC
EEF2
eRF1
eRF3
TIA1
AGO2
NPC
NPC
PABPC1
PABPC1
Calnexin
UNR*
UNR
PABPN1
EEF2
ERF1
ERF3
TIA1
GW182*
AGO2
10 µm
20 µm

S4 Fig. Subcellular localization of proteins in response to the CdtB of *Helicobacter hepaticus*.
Confocal imaging of Hep3B CdtB-expressing cells engrafted in mice (3 µm-tissue section) or transgenic CdtB-expressing cells* cultivated with doxycycline for 72 h was performed (as in Fig. 3A). Tissues/cells were processed for fluorescent staining with primary antibodies (associated with fluorescent-labeled secondary antibodies) generated against the proteins of interest (green) and NPC, calnexin, UNR or PABPC1 (red), depending on the origin of an antibody used to detect the protein of interest. Subsequent quantification of the proteins in nucleoplasm, cytoplasm and foci were performed using capture of fluorescent staining (confocal imaging) by measuring the pixel intensity with the "Plot Profile" function of ImageJ (v. 1.52n) [54], each count being performed on 100 NRs. The relative expression rate of protein in NR in response to the CdtB was reported as a fold increases versus the expression in the cytosol, with the exception of PABPN1 and GW182 absent in NR and calnexin due to lamellar staining. The discontinuous line shows the basal rate in the cytoplasm.
*Transgenic CdtB-expressing cells and ethanol permeabilization.

AGO2, Protein argonaute-2; C, cytoplasm; EEF2, eukaryotic elongation factor 2; eRF, eukaryotic release factors; GW182, trinucleotide repeat-containing gene 6A protein; IP3R2 and IP3R3, Inositol 1,4,5-trisphosphate receptor type 2 and 3; N, nucleus; NPC, Nuclear Pore Complex; PABPC1, cytoplasmic isoform of polyadenylate-binding protein 1; PABPN1, nuclear isoform of polyadenylate-binding protein 1; TIA1, T cell intracellular antigen; UNR, upstream of N-RAS.
