# Supplementary material for: Cytolethal distending toxin induces the formation of transient messenger-rich ribonucleoprotein nuclear invaginations in surviving cells
Source: PLoS Pathog. 2019 Sep 30;15(9):e1007921. doi: 10.1371/journal.ppat.1007921 (PMC6824578; doi:10.1371/journal.ppat.1007921)
Supplement: S5 Fig — Imaging of SW480 intestinal cells expressing the CdtB of H. hepaticus fused at its 3′ end to three repeats of the influenza hemagglutinin epitope (HA). Cells were processed for fluorescent staining with primary antibodies (associated with fluorescent-labeled secondary antibodies) generated against UNR (red) and the HA tag of the CdtB (red) as well as with DAPI to counterstain the nucleus (blue). Widefield and confocal imaging showed that CdtB did not colocalize with NR. (A) Widefield imaging showed that CdtB was detected mainly in the nucleus and excluded from the nucleoli (pink arrowheads), as expected [44]. (B) Confocal imaging showed that CdtB was detected in the cytoplasm, nucleus and excluded from the nucleoli. CdtB was also detected at the cell periphery lamellipodia and membrane ruffles (green arrows), as expected [44]. Subsequent quantification of the 3HA-tagged CdtB was performed using capture of fluorescent staining (confocal imaging) by measuring the pixel intensity with the “Plot Profile” function of ImageJ 1.51 [54], each count was performed on 100 cells. Yellow and pink arrowheads indicate UNR-NR and nucleoli, respectively. NR, nucleoplasmic reticulum. ns, not significant. (PDF) [file ppat.1007921.s005.pdf]

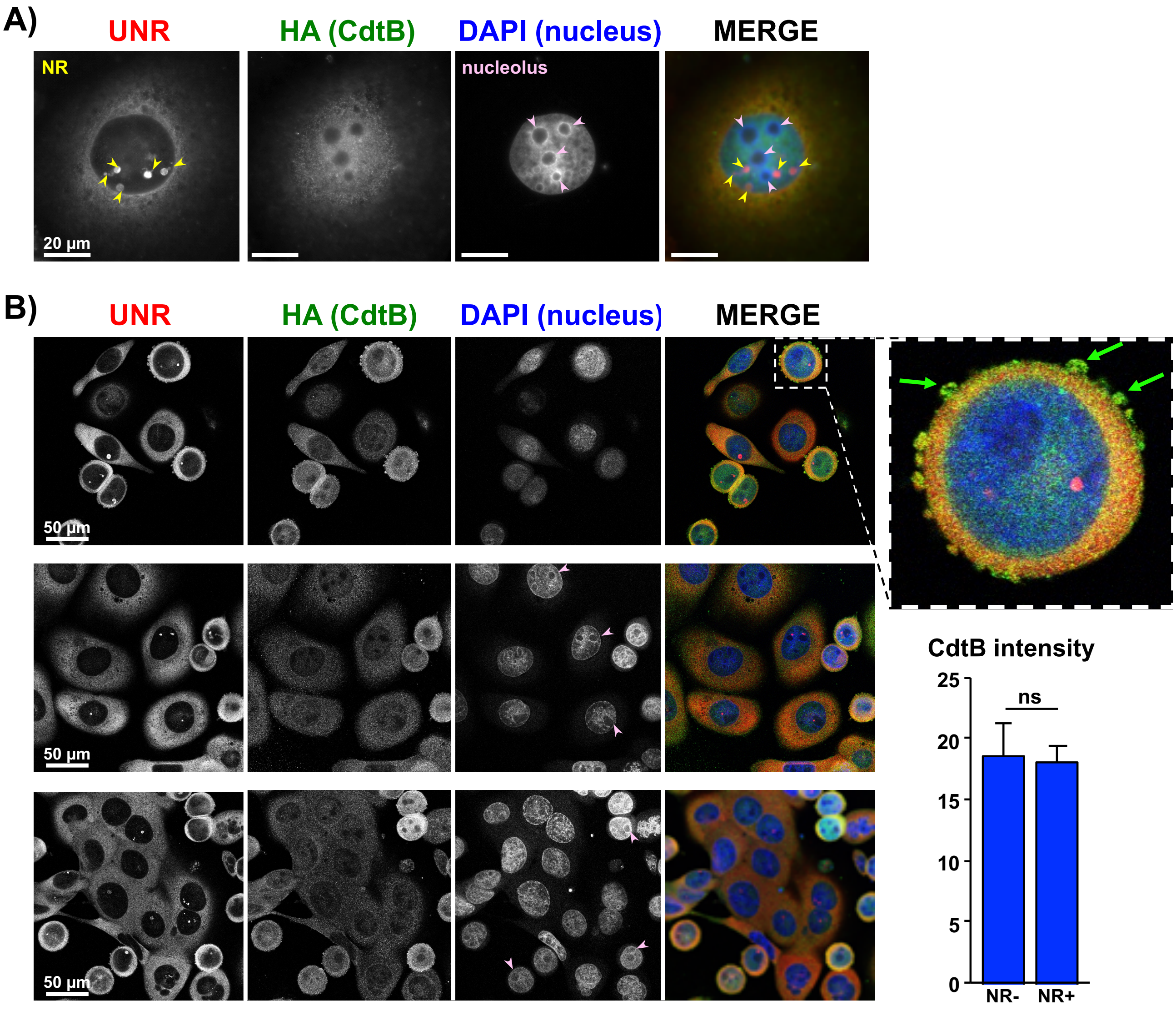

S5 Fig. Subcellular localization of proteins in response to the CdtB of *Helicobacter hepaticus* in intestinal cells.

Imaging of SW480 intestinal cells expressing the CdtB of *H. hepaticus* fused at its 3′ end to three repeats of the influenza hemagglutinin epitope (HA). Cells were processed for fluorescent staining with primary antibodies (associated with fluorescent-labeled secondary antibodies) generated against UNR (red) and the HA tag of the CdtB (red) as well as with DAPI to counterstain the nucleus (blue). Widefield and confocal imaging showed that CdtB did not colocalize with NR.

(A) Widefield imaging showed that CdtB was detected mainly in the nucleus and excluded from the nucleoli (pink arrowheads), as expected [44].

(B) Confocal imaging showed that CdtB was detected in the cytoplasm, nucleus and excluded from the nucleoli. CdtB was also detected at the cell periphery lamellipodia and membrane ruffles (green arrows), as expected [44]. Subsequent quantification of the 3HA-tagged CdtB was performed using capture of fluorescent staining (confocal imaging) by measuring the pixel intensity with the "Plot Profile" function of ImageJ 1.51 [54], each count was performed on 100 cells.

Yellow and pink arrowheads indicate UNR-NR and nucleoli, respectively.

NR, nucleoplasmic reticulum; ns, not significant.
