# Supplementary material for: Cytolethal distending toxin induces the formation of transient messenger-rich ribonucleoprotein nuclear invaginations in surviving cells
Source: PLoS Pathog. 2019 Sep 30;15(9):e1007921. doi: 10.1371/journal.ppat.1007921 (PMC6824578; doi:10.1371/journal.ppat.1007921)
Supplement: S6 Fig — 1- Bacterial genotoxin infection and cytoplasmic CdtB active subunit internalization 2- Giant nuclei together with profound nuclear reorganization in response to DNA damage—3- Cell cycle pause and DNA damage repair 4- Cell survival and cell cycle re-entry. (PDF) [file ppat.1007921.s006.pdf]

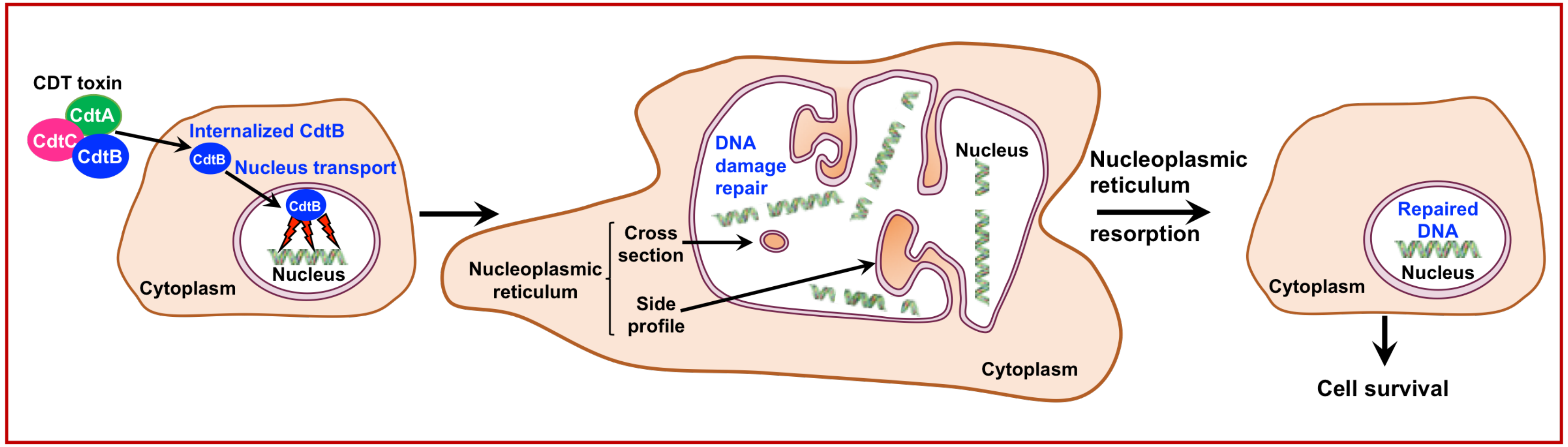

S6 Fig. Cytolethal distending toxin induces the formation of transient messenger-rich ribonucleoprotein nuclear invaginations in surviving cells

1- Bacterial genotoxin infection and cytoplasmic CdtB active subunit internalization

2- Giant nuclei together with profound nuclear reorganization in response to DNA damage -

3- Cell cycle pause and DNA damage repair

4- Cell survival and cell cycle re-entry
