# Supplementary material for: Cytolethal distending toxin induces the formation of transient messenger-rich ribonucleoprotein nuclear invaginations in surviving cells
Source: PLoS Pathog. 2019 Sep 30;15(9):e1007921. doi: 10.1371/journal.ppat.1007921 (PMC6824578; doi:10.1371/journal.ppat.1007921)
Supplement: S1 Table — (DOCX) [file ppat.1007921.s007.docx]

**Table S1. Antibodies used for immunohistochemistry and immunocytochemistry experiments**

| **Target protein** | **Clone, origin** | **Sourse/reference** | **Protein description from UniProtKB (except for HA, PMY and nuclear pore complex proteins)** | **Subcellular location** | **Dilution used** |
| --- | --- | --- | --- | --- | --- |
| AGO2  EIF2C2 | Monoclonal, 2E12-1C9, mouse | Abnova, M01 | Protein argonaute-2  Required for RNA-mediated gene silencing (RNAi) by the RNA-induced silencing complex (RISC). The 'minimal RISC' appears to include AGO2 bound to a short guide RNA such as a microRNA (miRNA) or short interfering RNA (siRNA). These guide RNAs direct RISC to complementary mRNAs that are targets for RISC-mediated gene silencing. The precise mechanism of gene silencing depends on the degree of complementarity between the miRNA or siRNA and its target. Binding of RISC to a perfectly complementary mRNA generally results in silencing due to endonucleolytic cleavage of the mRNA specifically by AGO2. Binding of RISC to a partially complementary mRNA results in silencing through inhibition of translation, and this is independent of endonuclease activity. May inhibit translation initiation by binding to the 7-methylguanosine cap, thereby preventing the recruitment of the translation initiation factor eIF4-E. May also inhibit translation initiation via interaction with EIF6, which itself binds to the 60S ribosomal subunit and prevents its association with the 40S ribosomal subunit. The inhibition of translational initiation leads to the accumulation of the affected mRNA in cytoplasmic processing bodies (P-bodies), where mRNA degradation may subsequently occur. In some cases RISC-mediated translational repression is also observed for miRNAs that perfectly match the 3' untranslated region (3'-UTR). Can also up-regulate the translation of specific mRNAs under certain growth conditions. Binds to the AU element of the 3'-UTR of the TNF (TNF-alpha) mRNA and up-regulates translation under conditions of serum starvation. Also required for transcriptional gene silencing (TGS), in which short RNAs known as antigene RNAs or agRNAs direct the transcriptional repression of complementary promoter regions. | Cytoplasm | 1/200 |
| c-FOS | Polyclonal, rabbit | Santa Cruz Biotechnology, sc-52 | Proto-oncogene c-Fos  Nuclear phosphoprotein which forms a tight but non-covalently linked complex with the JUN/AP-1 transcription factor. In the heterodimer, FOS and JUN/AP-1 basic regions each seems to interact with symmetrical DNA half sites. On TGF-beta activation, forms a multimeric SMAD3/SMAD4/JUN/FOS complex at the AP1/SMAD-binding site to regulate TGF-beta-mediated signaling. Has a critical function in regulating the development of cells destined to form and maintain the skeleton. It is thought to have an important role in signal transduction, cell proliferation and differentiation. In growing cells, activates phospholipid synthesis, possibly by activating CDS1 and PI4K2A. This activity requires Tyr-dephosphorylation and association with the endoplasmic reticulum | Nucleus | 1/100 |
| c-MYC | Polyclonal, A-14, rabbit | Santa Cruz Biotechnology, sc-789 | Myc proto-oncogene protein - Transcription factor that binds DNA in a non-specific manner, yet also specifically recognizes the core sequence 5'-CAC[GA]TG-3'. Activates the transcription of growth-related genes. Binds to the VEGFA promoter, promoting VEGFA production and subsequent sprouting angiogenesis | Nucleus | 1/100 |
| CANX | Polyclonal, rabbit | abcam, ab22595 | Calnexin - Calcium-binding protein that interacts with newly synthesized glycoproteins in the endoplasmic reticulum. It may act in assisting protein assembly and/or in the retention within the ER of unassembled protein subunits. It seems to play a major role in the quality control apparatus of the ER by the retention of incorrectly folded proteins. Associated with partial T-cell antigen receptor complexes that escape the ER of immature thymocytes, it may function as a signaling complex regulating thymocyte maturation. Additionally it may play a role in receptor-mediated endocytosis at the synapse. | Endoplasmic reticulum membrane | 1/100 |
| DDX6 | Rabbit, polyclonal | Novusbio, NB200-191 | Probable ATP-dependent RNA helicase DDX6.  In the process of mRNA degradation, plays a role in mRNA decapping (PubMed:16364915). Blocks autophagy in nutrient-rich conditions by repressing the expression of ATG-related genes through degration of their transcripts (PubMed:26098573) | Cytoplasm | 1/100 |
| EEF2 | Polyclonal, rabbit | abcam, ab40812 | Elongation factor 2 catalyzes the GTP-dependent ribosomal translocation step during translation elongation. During this step, the ribosome changes from the pre-translocational (PRE) to the post-translocational (POST) state as the newly formed A-site-bound peptidyl-tRNA and P-site-bound deacylated tRNA move to the P and E sites, respectively. Catalyzes the coordinated movement of the two tRNA molecules, the mRNA and conformational changes in the ribosome. | Cytoplasm | 1/100 |
| eIF4E | Monoclonal, 87/eIF-4E, mouse | BD biosciences, 610269 | Eukaryotic translation initiation factor 4E recognizes and binds the 7-methylguanosine-containing mRNA cap during an early step in the initiation of protein synthesis and facilitates ribosome binding by inducing the unwinding of the mRNAs secondary structures. Component of the CYFIP1-EIF4E-FMR1 complex which binds to the mRNA cap and mediates translational repression. In the CYFIP1-EIF4E-FMR1 complex this subunit mediates the binding to the mRNA cap | Cytoplasm | 1/100 |
| eIF4ENIF1, 4E-T | Polyclonal, goat | abcam, ab6034 | Eukaryotic translation initiation factor 4E transporter  Nucleoplasmic shuttling protein, which inhibits translation initiation. Mediates the nuclear import of EIF4E by a piggy-back mechanism. | Cytoplasm | 1/100 |
| eIF4G | Polyclonal, H-300, Rabbit | Santa Cruz Biotechnology, sc-11373 | Eukaryotic translation initiation factor 4G  Component of the protein complex eIF4F, which is involved in the recognition of the mRNA cap, ATP-dependent unwinding of 5'-terminal secondary structure and recruitment of mRNA to the ribosome. | Cytoplasm | 1/100 |
| eRF1, ETF1, TB3-1 RF1, SUP45L1 | Polyclonal, rabbit | [1] | Eukaryotic peptide chain release factor subunit 1  Directs the termination of nascent peptide synthesis (translation) in response to the termination codons UAA, UAG and UGA. Component of the transient SURF complex which recruits UPF1 to stalled ribosomes in the context of nonsense-mediated decay (NMD) of mRNAs containing premature stop codons. | Cytoplasm | 1/100 |
| eRF3, GSPT1 | Polyclonal, rabbit | [1] | Eukaryotic peptide chain release factor GTP-binding subunit ERF3A  Involved in translation termination in response to the termination codons UAA, UAG and UGA. Stimulates the activity of ERF1. Involved in regulation of mammalian cell growth. Component of the transient SURF complex which recruits UPF1 to stalled ribosomes in the context of nonsense-mediated decay (NMD) of mRNAs containing premature stop codons. | Cytoplasm | 1/100 |
| γH2AX | Phospho-Histone H2A.X (Ser139) (20E3) Rabbit | Cell Signaling, mAb #9718 | H2A histone family member X is a type of histone protein from the H2A family encoded by the H2AFX gene. In humans and other eukaryotes, the DNA is wrapped around histone octamers, consisting of core histones H2A, H2B, H3 and H4, to form chromatin. H2AX contributes to nucleosome-formation, chromatin-remodeling and DNA repair. H2AX becomes phosphorylated on serine 139, then called γH2AX, as a reaction on DNA double-strand breaks. | Nucleus | 1/100 |
| GW182 TNRC6, GW1, CAGH26, | Monoclonal, A-6, mouse | Santa Cruz Biotechnology, sc-374458 | Trinucleotide repeat-containing gene 6A protein plays a role in RNA-mediated gene silencing by both micro-RNAs (miRNAs) and short interfering RNAs (siRNAs). Required for miRNA-dependent repression of translation and for siRNA-dependent endonucleolytic cleavage of complementary mRNAs by argonaute family proteins. As scaffoldng protein associates with argonaute proteins bound to partially complementary mRNAs and simultaneously can recruit CCR4-NOT and PAN deadenylase complexes | Cytoplasm and P-body | 1/100 |
| HA | Monoclonal, 16B12, mouse | BioLegend Inc,  MMS-101R | The HA tag is derived from the human influenza hemagglutinin surface glycoprotein (corresponding to amino acids 98-106) of the human virus.  The product is reactive with CdtB-3HA HA-tagged fusion protein. | Tagged to the CdtB | 1/100 |
| hnRNP D,  AUF1 | Polyclonal, 07-260, rabbit | Millipore, 07-260 | Heterogeneous nuclear ribonucleoprotein D0 binds with high affinity to RNA molecules that contain AU-rich elements (AREs) found within the 3'-UTR of many proto-oncogenes and cytokine mRNAs. Also binds to double- and single-stranded DNA sequences in a specific manner and functions a transcription factor. Each of the RNA-binding domains specifically can bind solely to a single-stranded non-monotonous 5'-UUAG-3' sequence and also weaker to the single-stranded 5'-TTAGGG-3' telomeric DNA repeat. Binds RNA oligonucleotides with 5'-UUAGGG-3' repeats more tightly than the telomeric single-stranded DNA 5'-TTAGGG-3' repeats. Binding of RRM1 to DNA inhibits the formation of DNA quadruplex structure which may play a role in telomere elongation. May be involved in translationally coupled mRNA turnover. Implicated with other RNA-binding proteins in the cytoplasmic deadenylation/translational and decay interplay of the FOS mRNA mediated by the major coding-region determinant of instability (mCRD) domain. May play a role in the regulation of the rhythmic expression of circadian clock core genes. Directly binds to the 3'UTR of CRY1 mRNA and induces CRY1 rhythmic translation. May also be involved in the regulation of PER2 translation. AUF1 possesses 4 isoforms mainly nuclear and no antibody targeting specifically AUF1 cytosolic isoforms exist. | Predominantly in nucleus but is able to shuttle between nucleus and cytoplasm  (4 isoforms) | 1/100 |
| IP3R2, InsP3R2,ITPR3 | Polyclonal, C-20, goat | Santa Cruz Biotechnology, sc-7278 | Inositol 1,4,5-trisphosphate receptor type 2  Receptor for inositol 1,4,5-trisphosphate, a second messenger that mediates the release of intracellular calcium. This release is regulated by cAMP both dependently and independently of PKA (By similarity). | Endoplasmic reticulum membrane; Multi-pass membrane protein | 1/200 |
| IP3R3, InsP3R3, ITPR3 | Polyclonal, C-20, goat | Santa Cruz Biotechnology, sc-7277 | Inositol 1,4,5-trisphosphate receptor type 3  Receptor for inositol 1,4,5-trisphosphate, a second messenger that mediates the release of intracellular calcium. | Endoplasmic reticulum membrane; Multi-pass membrane protein | 1/200 |
| Ki-67 | Monoclonal, MIB-1, mouse | Dako  M7240 | The Ki-67 protein (also known as MKI67) is a nuclear protein that is associated with and may be necessary for cellular proliferation. During interphase, the Ki-67 antigen can be exclusively detected within the cell nucleus, whereas in mitosis most of the protein is relocated to the surface of the chromosomes. Ki-67 protein is present during all active phases of the cell cycle (G1, S, G2, and mitosis), but is absent in resting (quiescent) cells (G0). Cellular content of Ki-67 protein markedly increases during cell progression through S phase of the cell cycle. | Nucleus | 1/100 |
| Lamin A/C | Polyclonal, Guinea pig | gift from H. Wodrich  (Univ. Bordeaux) | Lamins are components of the nuclear lamina, a fibrous layer on the nucleoplasmic side of the inner nuclear membrane, which is thought to provide a framework for the nuclear envelope and may also interact with chromatin. Lamin A and C are present in equal amounts in the lamina of mammals. Plays an important role in nuclear assembly, chromatin organization, nuclear membrane and telomere dynamics. Required for normal development of peripheral nervous system and skeletal muscle and for muscle satellite cell proliferation. Required for osteoblastogenesis and bone formation. Also prevents fat infiltration of muscle and bone marrow, helping to maintain the volume and strength of skeletal muscle and bone. | Nuclar membrane | 1/500 |
| NSAP1, SYNCRIP,  hnRNP Q1,  hnRNP R-like protein | Polyclonal 07-260, rabbit | [2] | Heterogeneous nuclear ribonucleoprotein Q (hnRNP) implicated in mRNA processing mechanisms. Component of the CRD-mediated complex that promotes MYC mRNA stability. Isoform 1, isoform 2 and isoform 3 are associated in vitro with pre-mRNA, splicing intermediates and mature mRNA protein complexes. Isoform 1 binds to apoB mRNA AU-rich sequences. Isoform 1 is part of the APOB mRNA editosome complex and may modulate the postranscriptional C to U RNA-editing of the APOB mRNA through either by binding to A1CF (APOBEC1 complementation factor), to APOBEC1 or to RNA itself. May be involved in translationally coupled mRNA turnover. Implicated with other RNA-binding proteins in the cytoplasmic deadenylation/translational and decay interplay of the FOS mRNA mediated by the major coding-region determinant of instability (mCRD) domain. Interacts in vitro preferentially with poly(A) and poly(U) RNA sequences. Isoform 3 may be involved in cytoplasmic vesicle-based mRNA transport through interaction with synaptotagmins. Component of the GAIT (gamma interferon-activated inhibitor of translation) complex which mediates interferon-gamma-induced transcript-selective translation inhibition in inflammation processes. Upon interferon-gamma activation assembles into the GAIT complex which binds to stem loop-containing GAIT elements in the 3'-UTR of diverse inflammatory mRNAs (such as ceruplasmin) and suppresses their translation; seems not to be essential for GAIT complex function. | Cytoplasm | 1/200 |
| Nuclear Pore Complex Proteins | Monoclonal mAb 414, mouse | abcam, ab24609 | mAb 414, binds to nuclear pore complex proteins. | Nucleus membrane | 1/100 |
| PABPC1, PABP1 | Monoclonal 10E10, mouse | Sigma, P 6246 | Polyadenylate-binding protein 1 binds the poly(A) tail of mRNA, including that of its own transcript. May be involved in cytoplasmic regulatory processes of mRNA metabolism such as pre-mRNA splicing. Its function in translational initiation regulation can either be enhanced by PAIP1 or repressed by PAIP2. Can probably bind to cytoplasmic RNA sequences other than poly(A) in vivo. Involved in translationally coupled mRNA turnover. Implicated with other RNA-binding proteins in the cytoplasmic deadenylation/translational and decay interplay of the FOS mRNA mediated by the major coding-region determinant of instability (mCRD) domain. Involved in regulation of nonsense-mediated decay (NMD) of mRNAs containing premature stop codons; for the recognition of premature termination codons (PTC) and initiation of NMD a competitive interaction between UPF1 and PABPC1 with the ribosome-bound release factors is proposed. By binding to long poly(A) tails, may protect them from uridylation by ZCCHC6/ZCCHC11 and hence contribute to mRNA stability (PubMed:25480299). Positively regulates the replication of dengue virus (DENV) (PubMed:26735137). | Cytoplasm | 1/100 |
| PABPN1, PABP2 | Abgene EP3001Y | AJ1580B | Polyadenylate-binding protein 2  Involved in the 3'-end formation of mRNA precursors (pre-mRNA) by the addition of a poly(A) tail of 200-250 nt to the upstream cleavage product (By similarity). Stimulates poly(A) polymerase (PAPOLA) conferring processivity on the poly(A) tail elongation reaction and controls also the poly(A) tail length (By similarity). Increases the affinity of poly(A) polymerase for RNA (By similarity). Is also present at various stages of mRNA metabolism including nucleocytoplasmic trafficking and nonsense-mediated decay (NMD) of mRNA. Cooperates with SKIP to synergistically activate E-box-mediated transcription through MYOD1 and may regulate the expression of muscle-specific genes (PubMed:11371506). Binds to poly(A) and to poly(G) with high affinity (By similarity). May protect the poly(A) tail from degradation (By similarity). | Nucleus | 1/100 |
| PAIP1 | Monoclonal EPR13259, rabbit | abcam, ab181359 | Polyadenylate-binding protein-interacting protein 1Acts as a coactivator in the regulation of translation initiation of poly(A)-containing mRNAs. Its stimulatory activity on translation is mediated via its action on PABPC1. Competes with PAIP2 for binding to PABPC1. Its association with EIF4A and PABPC1 may potentiate contacts between mRNA termini. May also be involved in translationally coupled mRNA turnover. Implicated with other RNA-binding proteins in the cytoplasmic deadenylation/translational and decay interplay of the FOS mRNA mediated by the major coding-region determinant of instability (mCRD) domain. | Cytoplasm | 1/100 |
| PMY | Monoclonal, 12D10, mouse | Millipore MABE343 | Puromycin is an aminonucleoside antibiotic, derived from the *Streptomyces alboniger* bacterium, that functions as a protein synthesis inhibitor that blocks translation through premature chain termination in the ribosome.  This mAB detects puromycin incorporated into protein | Cytoplasm | 1/100 |
| RPL10A | Monoclonal, EPR12344, Rabbit | abcam, ab174318 | 60S ribosomal protein L10a  Component of the large ribosomal subunit. | Cytoplasm | 1/100 |
| TIA-1 | Monoclonal, 1h10,  mouse | [3] | Nucleolysin TIA-1 isoform p40  Involved in alternative pre-RNA splicing and regulation of mRNA translation by binding to AU-rich elements (AREs) located in mRNA 3' untranslated regions (3' UTRs). Possesses nucleolytic activity against cytotoxic lymphocyte target cells. May be involved in apoptosis | Nucleus, cytoplasmic, stress granules | 1/100 |
| TOM20 | Polyclonal, FL-145, rabbit | Santa Cruz Biotechnology, sc-11415 | Mitochondrial import receptor subunit TOM20  Central component of the TOM (translocase of outer membrane) receptor complex responsible for the recognition and translocation of cytosolically synthesized mitochondrial preproteins. Together with TOM22 functions as the transit peptide receptor at the surface of the mitochondrion outer membrane and facilitates the movement of preproteins into the TOM40 translocation pore. | Mitochondrial outer membrane protein | 1/100 |
| Unr, CSDE1 | Polyclonal, rabbit | Sigma, HPA018846 | Cold shock domain-containing protein E1  RNA-binding protein. Required for internal initiation of translation of human rhinovirus RNA. May be involved in translationally coupled mRNA turnover. Implicated with other RNA-binding proteins in the cytoplasmic deadenylation/translational and decay interplay of the FOS mRNA mediated by the major coding-region determinant of instability (mCRD) domain. | Cytoplasm | 1/100 |
| VTI1A | Polyclonal, 45/Vti1a, mouse | BD Biosciences, 611220 | Vesicle transport through interaction with t-SNAREs homolog 1A  V-SNARE that mediates vesicle transport pathways through interactions with t-SNAREs on the target membrane. These interactions are proposed to mediate aspects of the specificity of vesicle trafficking and to promote fusion of the lipid bilayers. Involved in vesicular transport from the late endosomes to the trans-Golgi network. Along with VAMP7, involved in an non-conventional RAB1-dependent traffic route to the cell surface used by KCNIP1 and KCND2. May be involved in increased cytokine secretion associated with cellular senescence. | Cytoplasm | 1/100 |

ICC immunocytochemistry

IF immunofluorescence.

References

1. Chauvin C, Salhi S, Le Goff C, Viranaicken W, Diop D, Jean-Jean O. Involvement of human release factors eRF3a and eRF3b in translation termination and regulation of the termination complex formation. Mol Cell Biol. 2005;25: 5801–5811. doi:10.1128/MCB.25.14.5801-5811.2005

2. Harris CE, Boden RA, Astell CR. A novel heterogeneous nuclear ribonucleoprotein-like protein interacts with NS1 of the minute virus of mice. J Virol. 1999;73: 72–80.

3. Taupin JL, Tian Q, Kedersha N, Robertson M, Anderson P. The RNA-binding protein TIAR is translocated from the nucleus to the cytoplasm during Fas-mediated apoptotic cell death. Proc Natl Acad Sci U S A. 1995;92: 1629–1633.
