# Supplementary material for: Cytolethal distending toxin induces the formation of transient messenger-rich ribonucleoprotein nuclear invaginations in surviving cells
Source: PLoS Pathog. 2019 Sep 30;15(9):e1007921. doi: 10.1371/journal.ppat.1007921 (PMC6824578; doi:10.1371/journal.ppat.1007921)
Supplement: S1 Methods — (PDF) [file ppat.1007921.s008.pdf]

Supplementary Materials and methods

Reagents and secondary antibodies

Doxycycline was purchased from Sigma Aldrich France. Alexa Fluor 647-labeled (far-red), Alexa Fluor 594-labeled (red), Alexa Fluor 488-labeled (green) secondary antibodies, Fluor 594-labeled phalloidin (red), and 4',6'-diamidino-2-phenylindole (DAPI, bleu) used for immunofluorescence were purchased from Molecular Probes (Eugene, OR, USA). ImmPRESS™ HRP anti-mouse IgG (peroxidase) polymer detection kit (Vector Laboratories, Laboratoires Eurobio/Abcys, Les Ulis, France) and EnVision+ System- HRP Labelled Polymer anti-rabbit (DAKO) were used as secondary antibodies for histology experiments.

Non-transgenic mice infection with *Helicobacter hepaticus*

Animal experiments were performed in A2 animal quarters (security level 2) by authorized personnel only. Before oral gavage, all mice were confirmed to be negative for the presence of *H. hepaticus* DNA in stools. Infection with *H. hepaticus* (strain 3B1) was performed at week 4 by oral gavage of 150 µL of *H. hepaticus* suspension in brucella broth on three successive days. Non-infected mice were treated similarly with brucella broth alone. Animals were fed an antioxidant-deprived diet (A04-10, SAFE) to minimize the anticarcinogenic effect of the diet. All mice infected by *H. hepaticus* were tested regularly and confirmed as being positive for the presence of *H. hepaticus* DNA in stools by a nested PCR specific to the *Helicobacter* genus, followed by DNA sequencing, as previously described [1][2]. Mice were sacrificed by cervical dislocation 14 months post-infection and colonization of the colon and liver was confirmed by nested PCR specific to the Helicobacter genus. Liver specimens were fixed in 3.7% formaldehyde solution in PBS, paraffin embedded, sectioned, and stained with hematoxylin–eosin. Slides were read blindly and interpreted by a histopathologist. Lesions were classified according to WHO criteria as focal cellular alteration (FCA), hepatocellular adenoma (AD), and hepatocellular carcinoma [3].

Ribosome-bound nascent chain puromycylation assay

To visualize newly synthetized proteins within cells, the ribopuromycylation (RPM) method was used as previously described [4]. Cells were grown on coverslips and incubated for 15 min at 37°C in complete medium supplemented with 208 µM emetin (EMD, Sigma). Cells were then treated with 355 µM cycloheximide (Sigma) for 2 min on ice in permeabilization buffer [50 mM Tris-HCl pH 7.5, 5 mM MgCl2, 25 mM KCl, 0.015% digitonin, EDTA-free protease inhibitor, 10 U/ml RNaseOut (Invitrogen)]. Cells were then washed and incubated on ice in polysome buffer (50 mM Tris-HCl pH 7.5, 5 mM MgCl2, 25 mM KCl, 0.2 M sucrose, EDTA-free protease inhibitor, 10 U/ml RNaseOut) supplemented with 91 µM puromycin (Sigma) for 10 min. After rapid washing in in polysome buffer, cells were fixed in 4%

formaldehyde for 15 min at room temperature. After fixation, cells were washed twice with PBS and immunostained with anti-puromycin antibody.

RT-qPCR experiments and quantification of the expression of the CSDE1 gene

Expression of CSDE1 gene encoding the UNR protein was measured by real time quantitative RT-qPCR with the forward (5'-GCACACCATTAATCCACTATGATCA-3') and reverse primers (5'-GGAGGGATGAAGAGGGAGATATTC-3') and normalized relative to the reference gene HPRT1, as previously reported [5]. Results are the means of three independent experiments, each performed in triplicate. Ratios were calculated using the ΔCt method.

Statistical analysis

Statistical analyses were performed using GraphPad Prism version 5 (GraphPad software, San Diego, CA, USA). The results are presented as the mean ± standard deviation. The means were compared using a non-parametric test; the Mann-Whitney or Wilcoxon test or Kruskal-Wallis, depending on the sampling, for comparison between two groups. A difference was considered significant when p was less than 0.05.

Supplementary references

1. Rocha M, Avenaud P, Ménard A, Le Bail B, Balabaud C, Bioulac-Sage P, et al. Association of *Helicobacter* species with hepatitis C cirrhosis with or without hepatocellular carcinoma. Gut. 2005;54: 396–401. https://doi.org/10.1136/gut.2004.042168 PMID: 15710989
2. Fox JG, Dewhirst FE, Shen Z, Feng Y, Taylor NS, Paster BJ, et al. Hepatic *Helicobacter* species identified in bile and gallbladder tissue from Chileans with chronic cholecystitis. Gastroenterology. 1998;114: 755–763. https://doi.org/10.1016/s0016-5085(98)70589-x PMID: 9516396
3. Deschl U, Cattley R, Harada T, Kuttler K, Hailey J, Hartig E, et al. Liver, Gallbladder, and Exocrine Pancreas. International Classification of Rodent tumors The Mouse. Mohr Ulrich; 2001. pp. 59–86. https://doi.org/10.1007/978-3-662-07973-7_3
4. David A, Dolan BP, Hickman HD, Knowlton JJ, Clavarino G, Pierre P, et al. Nuclear translation visualized by ribosome-bound nascent chain puromycylation. J Cell Biol. 2012;197: 45–57. https://doi.org/10.1083/jcb.201112145 PMID: 22472439
5. Péré-Védrenne C, Cardinaud B, Varon C, Mocan I, Buissonnière A, Izotte J, et al. The Cytolethal Distending Toxin Subunit CdtB of *Helicobacter* Induces a Th17-related and Antimicrobial Signature in Intestinal and Hepatic Cells *In Vitro*. J Infect Dis. 2016; 213: 1979–1989. https://doi.org/10.1093/infdis/jiw042 PMID: 26908757
