# Supplementary material for: Cytolethal distending toxin induces the formation of transient messenger-rich ribonucleoprotein nuclear invaginations in surviving cells
Source: PLoS Pathog. 2019 Sep 30;15(9):e1007921. doi: 10.1371/journal.ppat.1007921 (PMC6824578; doi:10.1371/journal.ppat.1007921)
Supplement: S1 Results — (PDF) [file ppat.1007921.s009.pdf]

Supplementary results

In vitro coculture experiments with *Helicobacter pylori* CagA+ and *Escherichia coli* STX2+

H. pylori is a CDT-negative bacteria that injects its CagA cytotoxin into the human gastric cell, triggering stress fiber formation and profound reorganization of the cell actin cytoskeleton leading to cell elongation, called "hummingbird" phenotype. *H. pylori* infection damages genomic and mitochondrial DNA in a reactive oxygen species (ROS)-dependent manner (reviewed in [1]) but it also directly damages host cell nuclear DNA [2]. As hepatic and intestinal cells could be resistant to *H. pylori* infection, Hep3B and SW480 coculture experiments were concomitantly performed using the *H. pylori*-susceptible AGS gastric cell line. Compared to non-infected cells, *H. pylori* infection did not reveal significant nuclear enlargement nor increase the number of UNR-NR in Hep3B and SW480 cells (S3 Fig), whatever the MOI, while elongated gastric AGS cells were observed at 24 h until 72 h of *H. pylori* infection (S3D Fig, blue arrowheads) but this cellular remodeling was not associated with an increase of UNR-NR. The increase of UNR-NRs was obvious in AGS cells during the infection with CDT- or colibactin-secreting bacteria (not shown and S3C Fig), some of these UNR-NR-positive infected AGS cells presented an elongated shape, reminiscent of the CagA-hummingbird phenotype. Cellular elongation, reminiscent of the CagA-associated hummingbird phenotype was also sometimes observed in SW480 cells (S3B Fig, blue arrowheads).

CDTs are trafficked in a retrograde manner from the cell surface through the Golgi apparatus and into the endoplasmic reticulum (ER) before ultimately reaching the host cell nucleus (reviewed in [3]). Some *E. coli* strains can secrete Shigatoxin that is similarly internalized into host cells and transported from the Golgi complex to the ER [4] and translocated to cytosolic targets. This results in host cell protein synthesis inhibition, activation of the ribotoxic stress response, the ER stress response, and, in some cases, the induction of apoptosis. As Shigatoxin triggers plasma membrane invaginations for its internalization in host cell [5], nuclear invagination formation was explored. Shigatoxin-2 secreting *E. coli* did not induce the increase in the number of UNR-NR in Hep3B, SW480 and AGS cells (S3A-C Fig), supporting that CdtB- and colibactin-induced nuclear DNA damage is associated with the NR formation.

SUPPLEMENTARY REFERENCES

1. Naumann M, Sokolova O, Tegtmeyer N, Backert S. *Helicobacter pylori*: A Paradigm Pathogen for Subverting Host Cell Signal Transmission. Trends Microbiol. 2017;25: 316–328. https//doi:10.1016/j.tim.2016.12.004 PMID: 28057411
2. Toller IM, Neelsen KJ, Steger M, Hartung ML, Hottiger MO, Stucki M, et al. Carcinogenic bacterial pathogen *Helicobacter pylori* triggers DNA double-strand breaks and a DNA damage response in its host cells. Proc Natl Acad Sci U S A. 2011;108: 14944–14949. https//doi::10.1073/pnas.1100959108 PMID: 21896770
3. DiRienzo JM. Uptake and processing of the cytolethal distending toxin by mammalian cells. Toxins. 2014;6: 3098–3116. https//doi::10.3390/toxins6113098 PMID: 25365527
4. Sandvig K, Garred O, Prydz K, Kozlov JV, Hansen SH, van Deurs B. Retrograde transport of endocytosed Shiga toxin to the endoplasmic reticulum. Nature. 1992;358: 510–512. https//doi: 10.1007/s00418-013-1111-z PMID: 1641040
5. Römer W, Berland L, Chambon V, Gaus K, Windschiegl B, Tenza D, et al. Shiga toxin induces tubular membrane invaginations for its uptake into cells. Nature. 2007;450: 670–675. https//doi:10.1038/nature05996 PMID: 18046403
